# Systematic review and meta-analysis of the acute effects of self-selected rest intervals on exercise performance maintenance, lactate levels, and heart rate

**DOI:** 10.1371/journal.pone.0354594

**Published:** 2026-07-24

**Authors:** LeTian Lv, Changxu Chen, Xuyang Liu, Huayang Liu, PanWen Wu

**Affiliations:** School of Physical Education, China West Normal University, Nanchong, China; Portugal Football School, Portuguese Football Federation, PORTUGAL

## Abstract

**Background:**

Rest interval duration is a key variable in exercise prescription, influencing both performance and physiological responses. Traditional training typically employs fixed rest intervals; however, this approach may not adequately reflect individual variability in recovery needs. Recently, self-selected rest intervals—based on individual perception—have gained attention as a potential strategy for more precise load regulation. Nevertheless, current evidence regarding their effects on exercise performance, heart rate, and blood lactate remains inconsistent.

**Methods:**

This study followed PRISMA guidelines and conducted a systematic search of Web of Science, PubMed, Cochrane Library, and Embase from database inception to March 20, 2026. Acute experimental studies comparing self-selected and fixed rest intervals were included. A three-level random-effects meta-analysis was performed to account for dependency among multiple effect sizes. Subgroup analyses, meta-regression, and sensitivity analyses were conducted to explore potential moderators and assess robustness.

**Results:**

Sixteen studies (k = 16) involving 281 participants were included. No significant overall difference was observed between self-selected and fixed rest intervals for exercise performance (g = 0.27, 95% CI [−0.14, 0.69], p = 0.29), with substantial between-study heterogeneity. After removing outliers, a small but significant effect favoring self-selected rest intervals was identified (g = 0.20, 95% CI [0.08, 0.33], p < 0.01). No significant differences were observed for heart rate (g = 0.002, p = 0.98) or blood lactate (g = −0.20, p = 0.36).

**Conclusion:**

Self-selected and fixed rest intervals produce comparable effects on exercise performance and physiological responses. However, self-selected rest intervals may confer a small advantage in maintaining performance under specific conditions. These findings should be interpreted with caution due to sensitivity to outliers and study heterogeneity.

Physical training refers to systematic, structured, and repetitive exercise interventions aimed at improving or maintaining an individual’s physical fitness level [1,2]. For adults, participating in physical training can maintain and improve physical health [3]; for athletes, appropriate physical training can significantly enhance athletic performance on the field [4–6]. Based on physiological adaptation characteristics, physical training includes various methods such as aerobic exercise, resistance training, and neuromuscular training (e.g., balance) [7]. In high-level training practices, training tasks often involve high intensity, intermittent, and irregular loads, posing significant challenges to the cardiovascular and neuromuscular systems. Consequently, there is growing emphasis on how to effectively regulate load and fatigue during training to maintain athletic performance and reduce fatigue accumulation [8].

Athletic performance and physiological responses (e.g., heart rate and blood lactate) are influenced by multiple interacting training variables, including intensity, volume, frequency, and recovery time [9]. Recent advances in training load regulation, such as repetitions in reserve (RIR) [10] and Velocity-Based Training (VBT) [11,12], have enabled more individualized and real-time adjustments of training intensity and volume.Meanwhile, rest interval duration also plays an important role in balancing fatigue and performance—although longer intervals may facilitate recovery, they may also reduce overall training stimulus [13]. However, in traditional practice, rest intervals are typically prescribed in a standardized manner (e.g., 1–3 minutes). Therefore, fixed rest strategies may not adequately account for inter- and intra-individual variability in fatigue, potentially affecting recovery and training adaptations [14].

In recent years, researchers have begun to incorporate the concept of “self-regulation” into interval duration settings, allowing trainees to autonomously determine recovery time based on their own perceptions (self-selected intervals) [15]. A large body of previous research has examined the effects of self-selected rest intervals on acute exercise performance during physical training.Some studies have reported beneficial effects on performance, which may be attributed to participants selecting longer rest periods, thereby facilitating neuromuscular recovery and maintaining higher levels of muscle contraction and force production capacity [16–18]. However, other studies have suggested that individuals’ perception-based assessment of recovery status may be subject to bias, and its accuracy remains uncertain. In addition, trainees may reduce rest duration in order to maintain training pace, potentially underestimating their recovery needs [19]. Therefore, whether self-selected rest intervals can serve as an effective alternative to fixed rest intervals remains to be further validated.

Given the inconsistent findings regarding self-selected rest intervals, different studies have yielded divergent conclusions regarding athletic performance and physiological responses. Furthermore, previous research has primarily consisted of small-sample studies lacking systematic integration, which limits the generalizability of the conclusions. Therefore, it is necessary to conduct a systematic review and meta-analysis to comprehensively evaluate the acute effects of self-selected versus fixed rest interval strategies.This study is the first to conduct a meta-analysis of randomized crossover trials to investigate the acute effects of self-selected rest intervals on exercise performance and physiological responses. Through subgroup analysis and regression analysis, it outlines potential factors that may influence exercise performance and physiological responses in the self-selected and fixed groups, enabling researchers and trainers to integrate relevant evidence and better manage training fatigue.

## 1. Materials and methods

### 1.1 Literature search

This study strictly adheres to the PRISMA guidelines for systematic reviews and meta-analyses: PRISMA for three-level meta-analysis [20], For the complete Prisma 2020 list, see S1 Appendix. Additionally, this review protocol was registered on PROSPERO on March 8, 2026 (registration number CRD420261334983). A systematic search was conducted in Web of Science, PubMed, Cochrane, and Embase, covering the period from the inception of each database to March 20, 2026. The search terms were (“self-selected rest” OR “self-selected recovery” OR “self-selected interval” OR “self-paced recovery” OR “self-regulated recovery” OR “autoregulated rest” OR “self-selected rest interval”) AND (“randomized controlled trial” OR randomized OR randomly OR trial). After the initial inclusion of studies, we conducted a reference tracing of the included studies to ensure comprehensiveness. The full search strategies for all databases are provided in S2 Appendix.

### 1.2 Literature screening strategy

One researcher (LT.Lv) independently used Zotero software to remove duplicate studies. Two other researchers (XY.Liu, CX.Chen) then reviewed the articles based on the inclusion and exclusion criteria. For studies that could not be excluded based on the abstract and title, the full text was read. In the event of any disagreement during the screening process, a third researcher (HX.Liu) was consulted for arbitration.

### 1.3 Inclusion and exclusion criteria

Inclusion and exclusion criteria were determined based on the PICOS framework. Inclusion criteria: (1) Population: Participants with at least one year of training experience; (2) Intervention: Use of self-selected rest intervals during training; (3) Comparison: All types of randomized controlled trials; (4) Outcomes: Measures must include at least one performance or physiological indicator, such as muscle strength or lactate levels; (5) Design: Acute original studies.Exclusion Criteria: (1) Literature types such as book chapters, opinion pieces, conference abstracts, and presentation abstracts; (2) Duplicate publications; (3) Publications for which the full text is unavailable; (4) Publications with unavailable or incomplete data.

### 1.4 Data extraction and conversion

Two researchers independently extracted data from the articles, including: (1) first author; (2) year of publication; (3) training design; (4) participant characteristics (number, gender, training level); (5) interval protocol design; (7) measurement results (average power, number of repetitions, average speed, lactate levels, heart rate). A third researcher reviewed the extracted data; in case of disagreement, a fourth researcher was consulted for arbitration. If data were presented as images, WebPlotDigitizer 4.1 was used for extraction [21]. If a study provided a confidence interval (CI), it was converted to a standard deviation (SD) [22]: SD = (CI_(high) – CI_(low))/ 2tN. If a study provided a standard error (SE), it was converted to an SD [22]: SD = N × √SE. Studies for which missing data could not be obtained were excluded from the analysis.

### 1.5 Assessment of study quality

Two researchers (XY.Liu, CX.Chen) independently assessed the risk of bias using the Cochrane Risk of Bias 2 tool, evaluating the following domains: randomization, deviation from the assigned intervention, missing outcome data, measurement of outcomes, and reporting of results [23]. Each assessment result was categorized as “low risk,” “high risk,” or “unclear.” In case of disagreement, a third researcher (HX.Liu) resolved the discrepancy.

### 1.6 Statistical analysis

Since some included studies reported multiple effect sizes (e.g., multiple treatment groups or multiple outcome measures), the use of a traditional two-level model may violate the assumption of independence of effect sizes [24]. Therefore, this study employed a three-level random-effects model for the meta-analysis [25]. This model decomposes the total variance into three levels: Level 1 (sampling variance), Level 2 (within-study variance), and Level 3 (between-study variance), thereby effectively addressing the interdependence of effects and the hierarchical data structure [26]. Additionally, the optimal model is determined by comparing the goodness-of-fit of the three-level model with that of the traditional two-level model, using the Akaike Information Criterion (AIC) and the Bayesian Information Criterion (BIC) [27].

Effect sizes were calculated as standardized mean differences (SMD) and adjusted using Hedges’ g. According to common criteria, values of 0.2, 0.5, and 0.8 represent small, medium, and large effects, respectively [26]. Model parameters were estimated using the restricted maximum likelihood (REML) method, and 95% confidence intervals (95% CI) were calculated [28]. Additionally, prediction intervals (PI) were calculated based on the t-distribution to provide supplementary information beyond the confidence intervals for random-effects models [29,30].

Heterogeneity was assessed using the I^2^ statistic, with the following interpretation criteria: 0–30% indicates low heterogeneity, 31–50% indicates moderate heterogeneity, 51–75% indicates high heterogeneity, and 76–100% indicates very high heterogeneity [31]. To further enhance the robustness of the estimates, this study employed robust variance estimation (RVE) and implemented it using the clubSandwich R package. In the RVE analysis, the within-study correlation coefficient was initially set to 0.6, with sensitivity analyses conducted for values of 0.4 and 0.8.

Following a previous participant classification framework [32], participants were categorized as “athletes” (training/developing, well-trained/national level, elite/international level, world-class) and “non-athletes” (recreationally active).To better explain potential differences in athletic performance across different types, outcome measures were categorized into four groups based on exercise physiology theory and prior research [33,34]: strength/power, anaerobic performance, muscular endurance, and aerobic endurance. Specific classifications were based on exercise duration, primary energy systems, and neuromuscular characteristics.

Subgroup analysis and meta-regression were employed to explore potential moderating factors, with categorical variables analyzed via subgroup analysis and continuous variables via meta-regression. Publication bias was assessed using funnel plots and the Egger test; when test results indicated potential bias, the trim-and-fill method was used for correction [35].

Sensitivity analyses were conducted within a three-level model framework, including: (1) altering correlation coefficients to assess their impact on standard errors; (2) performing leave-one-out analysis by sequentially excluding studies; (3) identifying potential outliers and high-impact studies based on model diagnostics, using standardized residuals for evaluation; studies with absolute standardized residuals greater than 2 were classified as potential outliers [36]. After excluding outliers, the model was re-estimated to assess the robustness of the results.

All statistical analyses were performed in R 4.5.1, primarily using the metafor package for analysis and ggplot2 for result visualization. The code used to generate the results and figures is available at: https://github.com/lotte559595/meta.

### 1.7 Assessment of evidence certainty

The GRADE methodology [37] was used to interpret study findings in conjunction with the results of the risk of bias assessment, and the certainty of the evidence was rated as “high,” “moderate,” “low,” or “very low.” One researcher independently performed the GRADE assessment, and another researcher verified the results.

## 2. Results

### 2.1 Results of the literature search

**Fig 1:**
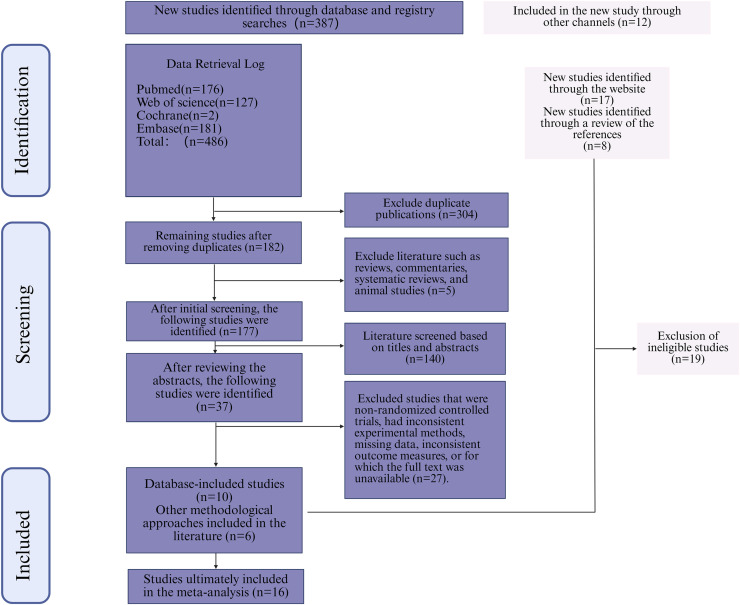
PRISMA flow diagram of study selection.

A systematic search of PubMed, Web of Science, Cochrane Library, and Embase identified 486 records. After screening titles, abstracts, and full texts according to the predefined inclusion and exclusion criteria, 16 studies were ultimately included. Detailed characteristics are provided in Fig 1.

### 2.2 Basic characteristics of included studies

A total of 16 studies were included in this review. Among them, one study by Roberto Simão et al. employed a randomized controlled trial design [38], whereas the remaining studies used randomized crossover designs [15,39–41,42–51].

In total, 281 participants were included (235 males and 46 females), with sample sizes ranging from 12 to 33 per study. All studies incorporated self-selected rest intervals. Among these, four studies [38,41,43,46] compared multiple fixed rest durations, whereas 12 studies [39,40,42,44,45,47–51] used a single fixed interval as the control condition.

Regarding exercise modality, eight studies [38–41,43,47,50,51] employed resistance training protocols, while the remaining studies [15,42–45,47,48] used high-intensity interval training (HIIT). Fixed rest intervals ranged from 1 to 3 minutes in resistance training and from 30 to 90 seconds in HIIT protocols.

Additionally, four studies [15,41,42,44] used auxiliary scales to guide participants in determining rest duration, whereas the remaining studies relied solely on subjective perception. Detailed characteristics are provided in S3 Appendix.

### 2.3 Risk of bias assessment

Only two studies [40,41] clearly described the randomization process and were therefore classified as low risk of bias. One study [38] presented concerns regarding the randomization process.

**Fig 2:**
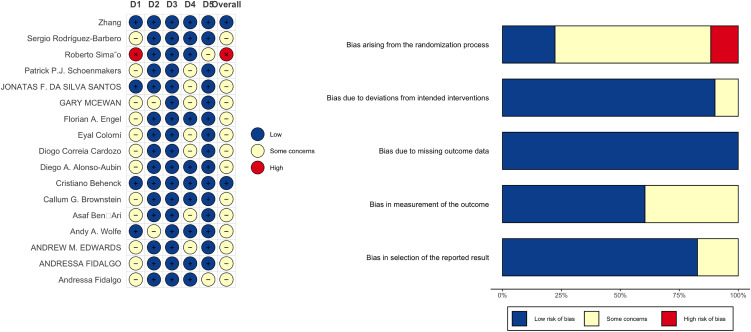
Risk of bias assessment of included studies.

Most studies provided detailed descriptions of the intervention protocols and employed objective outcome measures; thus, the risk of bias related to deviations from intended interventions and outcome measurement was generally rated as low. Overall, all included studies exhibited at least some concerns regarding risk of bias. Detailed assessments are presented in Fig 2.

### 2.4 Meta-analysis of exercise performance

**Fig 3:**
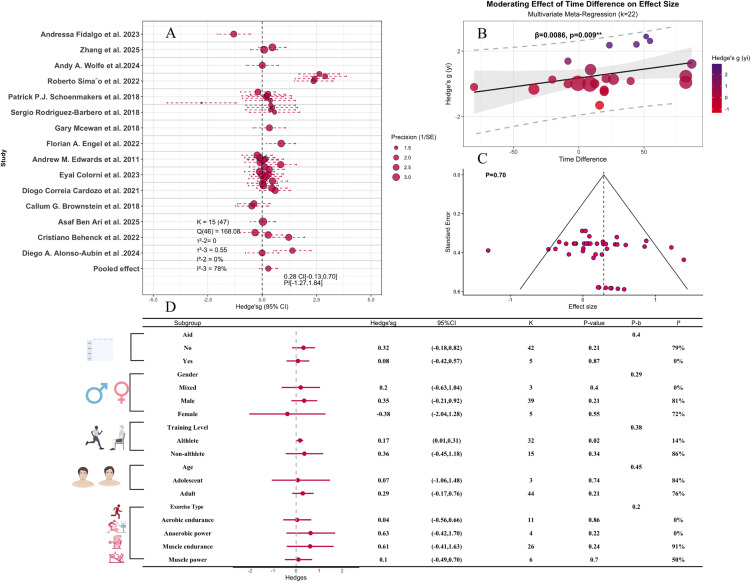
Forest plot of the meta-analysis for exercise performance maintenance. ***Notes: K*,** the total number of effects included in the pooled effect size; ***Hedge’s g***, the effect size indicators used in the pooled; ***95%CI***, 95% confidence interval; ***P-b***, statistically significant P values for pooled effect between moderator; ***P-value***, statistically significant P values for specific pooled effect of moderator; ***I***^**2**^, quantitative indicators of heterogeneity; ***β***, represent the slopes. ***t***^***2***^***-2***, within-study variance; ***t***^***2***^***-3,*** between-study variance; ***I***^**2**^**-2,** within-study heterogeneity; ***I***^**2**^**-3,** between-study heterogeneity.

The overall meta-analysis revealed no statistically significant difference between self-selected and fixed rest intervals on exercise performance outcomes (k = 47, g = 0.27, 95% CI [−0.14, 0.69], p = 0.29). Heterogeneity analysis indicated minimal within-study variance (I² Level 2 = 1%) but substantial between-study heterogeneity (I² Level 3 = 78%), with a wide prediction interval (PI = [−1.30, 1.85]) (GRADE = Very Low), suggesting considerable variability in the true effects across studies (Fig 3 and S9 Appendix).

According to the criteria proposed by Hunter and Schmidt [52], when sampling variance accounts for less than 75% of total variance, substantial true heterogeneity is present. In this study, sampling variance accounted for only 21.04%, indicating that the observed variability in effect sizes cannot be explained solely by sampling error.

Furthermore, variance decomposition showed that more than 98% of total heterogeneity originated from between-study differences, whereas within-study variance was negligible. These findings justified further moderator analyses to explore potential sources of heterogeneity.

#### 2.4.1 Moderator analysis for exercise performance.

Subgroup analyses showed that between-subgroup differences were not statistically significant for any moderator variables (p > 0.05), suggesting that these factors did not significantly explain the overall effect size. A significant effect was observed only within the athlete subgroup (p < 0.05).

Meta-regression analysis indicated that time difference significantly moderated the effect size (β = 0.0086, p = 0.0092), Meta-regression revealed that time difference significantly moderated effect size (β = 0.0086, p = 0.0092), indicating a positive association between greater rest interval differences and performance effects.

#### 2.4.2 Publication bias for exercise performance.

No evidence of publication bias was observed based on funnel plot symmetry and Egger’s test (p = 0.70).

### 2.5 Meta-analysis of heart rate

**Fig 4:**
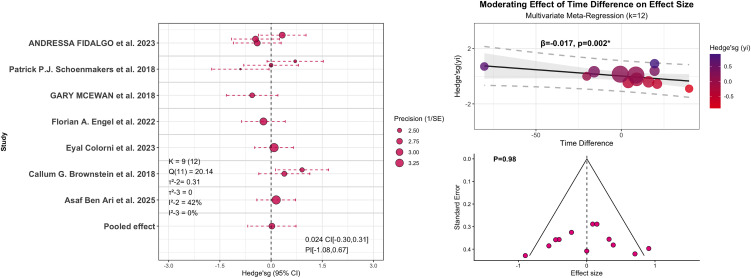
Meta-analysis results for heart rate. Notes: ***K***, the total number of effects included in the pooled effect size; ***Hedge’s g***, the effect size indicators used in the pooled; ***95%CI***, 95% confidence interval; ***P-value***, statistically significant P values for specific pooled effect of moderator; ***β***, represent the slopes.***t***^***2***^***-2***, within-study variance;***t***^***2***^***-3***, between-study variance; ***I***^***2***^***-2***, within-study heterogeneity; ***I***^***2***^***-3***, between-study heterogeneity.

No significant difference was found between conditions for post-exercise heart rate (k = 12, g = 0.00, 95% CI [−0.30, 0.31], p = 0.98). Moderate within-study heterogeneity was observed (I^2^ Level 2 = 42%), with no between-study heterogeneity (I^2^ Level 3 = 0%) and a prediction interval of [−0.76, 0.77] (GRADE = Low) (Fig 4 and S9 Appendix).

#### 2.5.1 Moderator analysis for heart rate.

No significant subgroup differences were found (p > 0.05). Meta-regression indicated that time difference significantly moderated effect size (β = −0.017, p = 0.002), with larger differences associated with reduced heart rate effects.detailed characteristics are provided in s4 appendix.

#### 2.5.2 Publication bias for heart rate.

The funnel plot appeared symmetrical, and Egger’s test was not statistically significant (p = 0.98), indicating no evidence of publication bias.

### 2.6 Meta-analysis of blood lactate

**Fig 5:**
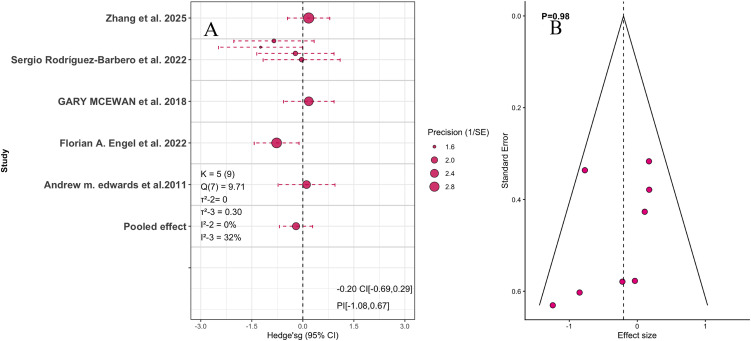
Meta-analysis results for blood lactate. Notes: ***K***, the total number of effects included in the pooled effect size; ***Hedge’s g***, the effect size indicators used in the pooled; ***95%CI***, 95% confidence interval; ***P***, statistically significant P values for specific pooled effect of moderator; ***t***^***2***^***-2***, within-study variance; ***t***^***2***^***-3***, between-study variance; ***I***^***2***^***-2***, within-study heterogeneity; ***I***^***2***^***-3***, between-study heterogeneity.

No significant difference was found between self-selected and fixed rest intervals in blood lactate responses (k = 9, g = −0.20, 95% CI [−0.69, 0.29], p = 0.36). Heterogeneity was low (I² Level 2 = 0%, I² Level 3 = 32%), with a prediction interval of [−1.08, 0.67] (GRADE = Low) (Fig 5 and S9 Appendix).

#### 2.6.1 Moderator analysis for blood lactate.

No significant between-group differences were observed (p > 0.05). A significant effect was found only within the female subgroup (p < 0.05). Detailed characteristics are provided in S4 Appendix.

Meta-regression indicated that time difference was not a significant moderator (p > 0.05).

### 2.7 Sensitivity analysis for exercise performance

Sensitivity analyses using different assumed correlation coefficients (r = 0.4, 0.6, 0.8) produced consistent results. Detailed characteristics are provided in S6 Appendix.

Outlier diagnostics identified high-influence cases (e.g., studies by Andressa Fidalgo et al. and Roberto Simão et al.). After excluding these studies, the effect became statistically significant (k = 41, g = 0.20, 95% CI [0.08, 0.33], p < 0.01), with substantially reduced heterogeneity (I² Level 2 = 4.2%, I² Level 3 = 0%).

However, the prediction interval (PI = [−0.01, 0.41]) still indicated variability in true effects across studies.

Subgroup analyses indicated significant effects in studies without auxiliary scales, in adults, and in males, but not in females or mixed samples. Meta-regression results remained significant (β = 0.0091, p = 0.002). Leave-one-out analyses confirmed robustness. Detailed characteristics are provided in S5–S8 Appendix.

### 2.8 Sensitivity analysis for heart rate

Results were stable across correlation assumptions. One influential study (Callum G. Brownstein et al.) was identified. After exclusion, results remained non-significant (k = 11, g = −0.06, p = 0.60), with minimal changes. Leave-one-out analyses confirmed robustness. Detailed characteristics are provided in S5–S8 Appendix.

### 2.9 Sensitivity analysis for blood lactate

Results were consistent across correlation assumptions. No influential outliers were detected, and leave-one-out analyses confirmed stability of pooled estimates. Detailed characteristics are provided in S5–S8 Appendix.

## 3. Discussion

This systematic review and meta-analysis comprehensively evaluated the acute effects of self-selected rest intervals on exercise performance, heart rate, and blood lactate responses. To our knowledge, this is the first study to systematically synthesize evidence on self-selected interval strategies within a unified analytical framework. Importantly, by employing a three-level meta-analytic model, this study effectively accounted for dependency among multiple effect sizes within studies, thereby enhancing the methodological rigor and reliability of the findings.

Overall, no statistically significant differences were observed between self-selected and fixed rest intervals in terms of exercise performance, heart rate, or blood lactate. However, sensitivity analyses indicated that the pooled effect for exercise performance was influenced by a small number of outlying studies. After excluding these studies, a small statistically effect favoring self-selected rest intervals was observed. These findings suggest that the estimated effect may be sensitive to influential studies and should be interpreted with caution.

Further moderator analyses indicated that factors such as gender, training level, age, and the use of supplementary scales may influence the observed effects. Nevertheless, these findings were not entirely stable across sensitivity analyses, and the prediction intervals consistently included zero, indicating substantial variability in true effects across studies. Therefore, the potential advantages of self-selected rest intervals should be interpreted with caution.

### 3.1 Maintenance of exercise performance

The overall meta-analysis indicated no significant difference between self-selected and fixed rest intervals in maintaining exercise performance across both resistance and non-resistance training modalities. However, after excluding outliers, self-selected rest intervals demonstrated a statistically significant advantage, suggesting that influential studies substantially affected the pooled estimates.

Closer examination of the identified outliers suggests that they may reflect distinct underlying mechanisms. In the study by ANDRESSA FIDALGO et al., the self-selected rest interval (14.04 s) was markedly shorter than the fixed interval (30 s). Under high-intensity resistance circuit training conditions, energy supply is primarily dependent on the phosphagen system, which typically requires approximately 30–60 seconds or longer for adequate recovery [53,54]. In practice, individuals may prioritize maintaining training rhythm and initiate subsequent sets based on subjective readiness, leading to shorter rest intervals [55]. Such insufficient recovery may result in increased metabolic accumulation and neuromuscular fatigue, thereby impairing subsequent performance.

In contrast, the study by Roberto Simão et al. reported a significant advantage of self-selected intervals. Notably, participants in the self-selected condition also chose longer rest intervals in this study. In addition, this study employed a parallel randomized controlled design, in contrast to the crossover designs used in most other included studies. Compared with crossover designs, parallel designs are less effective in controlling inter-individual variability in factors such as strength, endurance, and recovery capacity [56,57]. Under self-regulated conditions, such variability may be further amplified, potentially contributing to the observed differences.

Taken together, the identified outliers likely reflect both insufficient physiological recovery and methodological variability. This highlights that the effectiveness of self-selected rest intervals is not uniform and may depend on the interaction between subjective regulation and physiological demands.

### 3.2 Moderators of performance maintenance

Subgroup analyses did not reveal statistically significant differences between subgroups, suggesting that demographic variables may have limited influence on the overall effect. However, a significant effect was observed within the athlete subgroup, indicating that trained individuals may derive greater benefit from self-selected strategies.

From a mechanistic perspective, athletes typically possess greater training experience and enhanced sensitivity to fatigue, enabling more accurate self-regulation of recovery. This interpretation aligns with previous findings on autoregulation strategies, including RIR and velocity-based approaches [58,59]. Furthermore, the validity of perceptual measures such as RPE has been shown to depend on training experience, supporting the notion that experienced individuals can more effectively utilize subjective feedback.

After removing outliers, additional moderators (e.g., auxiliary scale use, gender, and training level) became statistically significant. This suggests that influential studies may obscure underlying moderator effects. Interestingly, studies without auxiliary scales demonstrated significant effects, which may indicate that fully self-regulated strategies better reflect internal physiological states. Conversely, external scales may constrain decision-making, leading to more conservative recovery strategies.

Gender differences were also observed, with significant effects in males but not females. This may reflect sex-specific physiological characteristics, such as greater fatigue resistance and metabolic stability in females [60]. However, given the limited number of female participants, this finding should be interpreted cautiously.

Importantly, meta-regression consistently showed that the difference in rest interval duration between conditions significantly moderated the effect size. This finding suggests that performance outcomes are primarily driven by the magnitude of recovery time differences, rather than the regulatory strategy itself. From a physiological perspective, recovery processes—such as phosphagen resynthesis and metabolite clearance—are time-dependent, supporting this interpretation.

Overall, these findings indicate that the effectiveness of rest interval strategies is likely determined by actual recovery duration rather than whether intervals are self-selected or fixed.

### 3.3 Heart rate and blood lactate

No significant differences were observed between self-selected and fixed rest intervals in heart rate or blood lactate responses. These findings remained stable even after sensitivity analyses, suggesting that rest interval strategy has limited influence on these physiological markers.

Meta-regression indicated that longer self-selected intervals were associated with slightly lower heart rate responses; however, this effect was small and did not translate into a significant overall difference. This finding aligns with previous research suggesting that heart rate is primarily influenced by overall training load rather than interval structure [61].

Additionally, the “heart rate lag” phenomenon may reduce sensitivity to short-term changes in interval duration, resulting in relatively stable heart rate responses during exercise. In resistance training, similar findings have been reported, indicating that heart rate is more strongly influenced by exercise characteristics than rest duration [62].

Similarly, no significant differences were observed in blood lactate responses. This may be related to a compensatory mechanism, whereby shorter rest intervals may increase lactate accumulation while simultaneously reducing subsequent exercise performance or total work, resulting in comparable overall metabolic stress [63,64].

Although a significant effect was observed in the female subgroup, this did not translate into overall significance and may reflect small sample sizes or study-specific factors. Furthermore, the lack of detailed reporting on interval durations limits interpretation of this finding.

Collectively, these results suggest that heart rate and blood lactate may have limited utility as indicators of local fatigue or recovery adequacy in this context.

### 3.4 Limitations

Several limitations should be acknowledged. First, the number of included studies was relatively small, and most studies had limited sample sizes, potentially reducing statistical power. Second, the predominance of crossover designs may introduce order and learning effects, while the limited number of parallel trials restricts generalizability.

Third, this study focused on acute responses and cannot infer long-term training adaptations. Fourth, the low proportion of female participants limits conclusions regarding sex differences. Finally, the classification of exercise modalities based on energy systems may overlook sport-specific characteristics.

Future studies should employ larger sample sizes, standardized protocols, and clearer definitions of self-selected intervals to improve evidence quality.

### 3.5 Future research directions

Future research should focus on improving the regulation of self-selected rest intervals. Current approaches rely heavily on subjective perception, which may not accurately reflect physiological recovery. Integrating objective performance metrics (e.g., repetitions, velocity, power output) may improve reliability.

Additionally, developing multi-parameter regulation models that combine subjective, physiological, and neuromuscular indicators may provide a more comprehensive assessment of recovery.

More high-quality randomized controlled trials are needed to validate these findings and improve external validity. Future studies should also examine different populations and investigate long-term training adaptations associated with self-selected strategies.

### 3.6 Practical applications

The findings suggest that the distinction between self-selected and fixed rest intervals may be less important than the actual recovery duration achieved. Therefore, training practices should shift toward performance-based regulation of rest intervals.

Coaches and practitioners may benefit from adjusting rest intervals based on performance indicators (e.g., repetitions, velocity, or power output), rather than relying solely on subjective perception or physiological markers. This approach may enhance precision in load management and improve training outcomes.

## 4. Conclusion

This study evaluated the acute effects of self-selected rest intervals on exercise performance and physiological responses using a systematic review and three-level meta-analysis. Overall, no significant differences were observed between self-selected and fixed rest intervals in exercise performance, heart rate, or blood lactate.

However, after excluding potential outliers, self-selected rest intervals demonstrated a small but significant advantage in maintaining performance, suggesting that their effectiveness may depend on specific conditions.

Importantly, the results indicate that training outcomes are more strongly influenced by actual recovery duration than by whether rest intervals are self-selected or fixed. Therefore, future research and practice should prioritize performance-based approaches to rest interval regulation to optimize training load management.

## Supporting information

S1 Appendix PRISMA 2020 checklist. (DOCX)

S2 Appendix Searching strategies for various databases. (DOCX)

S3 Appendix Table of study characteristics. (DOCX)

S4 Appendix Subgroup analysis table for blood lactate and heart rate. (DOCX)

S5 Appendix Sensitivity analysis. (DOCX)

S6 Appendix Robust variance estimation. (DOCX)

S7 Appendix Main effects plot with outliers removed. (DOCX)

S8 Appendix Subgroup analysis after outlier removal. (DOCX)

S9 Appendix Summary of evidence quality. (DOCX)

S10 Appendix Regression analysis with outlier removal. (DOCX)
