# Supplementary material for: Systematic review and meta-analysis of the acute effects of self-selected rest intervals on exercise performance maintenance, lactate levels, and heart rate
Source: PLoS One. 2026 Jul 24;21(7):e0354594. doi: 10.1371/journal.pone.0354594 (PMC13399479; doi:10.1371/journal.pone.0354594)
Supplement: S2 Appendix — (DOCX) [file pone.0354594.s002.docx]

**Electronic Supplementary Material Appendix S2**

Searching strategies for various databases

| Database | Searching Various Databases |
| --- | --- |
| Pubemed | (("Rest Periods"[MeSH Terms] OR "recovery"[MeSH Terms] OR rest[Title/Abstract] OR "rest interval"[Title/Abstract] OR "recovery interval"[Title/Abstract] OR "recovery time"[Title/Abstract]) AND  ("self-selected"[Title/Abstract] OR "self regulated"[Title/Abstract] OR self-regulated[Title/Abstract] OR autoregulated[Title/Abstract] OR"self-paced"[Title/Abstract]OR"perceivedrecovery"[Title/Abstract])AND("randomized controlled trial"[Publication Type] OR randomized[Title/Abstract] OR randomly[Title/Abstract] OR crossover[Title/Abstract] OR trial[Title/Abstract])) |
| Web of science | TS=(("rest interval" OR "rest periods" OR "recovery interval" OR "recovery time" OR recovery)AND("self-selected" OR "self regulated" OR self-regulated OR autoregulated OR "self-paced" OR "perceived recovery")AND("randomized controlled trial" OR randomized OR randomly OR crossover OR trial)) |
| Embase | ('rest period'/exp OR 'recovery'/exp OR 'rest interval' OR 'recovery interval' OR 'recovery time')AND('self regulation'/exp OR 'self selected' OR 'self-paced' OR 'autoregulation' OR 'perceived recovery')AND('randomized controlled trial'/exp OR random*:ti,ab OR crossover:ti,ab OR trial:ti,ab) |
| Cochrane | (MeSH descriptor: [Rest] OR MeSH descriptor: [Recovery] OR "rest interval" OR "recovery interval")AND("self-selected" OR "self regulated" OR autoregulated OR "self-paced")ANDMeSH descriptor: [Randomized Controlled Trial] OR randomized OR crossover OR trial) |
