# Supplementary material for: Systematic review and meta-analysis of the acute effects of self-selected rest intervals on exercise performance maintenance, lactate levels, and heart rate
Source: PLoS One. 2026 Jul 24;21(7):e0354594. doi: 10.1371/journal.pone.0354594 (PMC13399479; doi:10.1371/journal.pone.0354594)
Supplement: S3 Appendix — (DOCX) [file pone.0354594.s003.docx]

**Electronic Supplementary Material Appendix S3 (Table of Study Characteristics)**

| First author |  |  | Characteristics of the Study Population | | | Outcome Measures |  |  |  |  |
| --- | --- | --- | --- | --- | --- | --- | --- | --- | --- | --- |
|  | Year of Publication (Year) | Group | Physical Activity Level | Gender  (Male/Female) | Age (Mean ± SD) | Research Area | Training Method | Workload intensity | Fixed rest intervals | Use of Assistive Devices Select Custom Time |
| ANDRESSA FIDALGO | 2023 | SS ri (n=16)  Fixed ri (n=16) | Healthy males | 16/0 | 27.1 ± 3.9 | ① | High-intensity resistance training | 20s peak stimulus | 30s | No |
| Zhang | 2025 | SS ri (n=20)  Fixed ri (n=20) | Young people | 11/9 | 23.2 ± 3.1 | ①③ | Resistance training | 75% 1RM | 3 min | No |
| Andy A. Wolfe | 2024 | SS ri (n=14)  Fixed ri (n=14) | Young men | 7/7 | 20.6 ± 1.65 | ① | Resistance training | 80% 1RM | 2 min | Yes |
| Roberto Sima˜o | 2022 | SS ri (n=16)  Fixed ri(n=17) | Young men | 33/0 | 21.8 ± 2.3 | ① | Resistance training | NA | 75 s | No |
| Patrick P.J. Schoenmakers | 2018 | SS ri (n=12)  Fixed1 ri (n=12)  Fixed2 ri (n=12)  Fixed3 ri (n=12) | Male runners participating in recreational training  Male runners | 12/0 | 34±11 | ① | High-intensity interval training | 92.5% V̇O2max | 1 min  2 min  3 min | Yes |
| Sergio Rodríguez-Barbero | 2022 | SS ri (n=12)  Fixed1 ri(n=12)  Fixed2 ri (n=12) | High-level middle- and long-distance running  Athletes | 6/6 | 23.82 ± 7.83 | ①③ | High-intensity interval training | ~ 90% of their  personal best | 1 min  2 min | No |
| GARY MCEWAN | 2018 | SS ri (n=14)  Fixed ri (n=14) | Men with training experience | 14/0 | 30 ± 7 | ①③ | High-intensity interval training | 105% MAS | 30s | No |
| Andressa Fidalgo | 2023 | SS ri (n=16)  Fixed10 ri (n=16)  Fixed30 ri (n=16) | Well-trained men | 16/0 | 27.1 ± 3.9 | ② | High-intensity resistance training | 20s peak stimulus | 10s  30s | Yes |
| Florian A. Engel | 2022 | SS ri(n=19)  Fixed ri (n=19) | Youth soccer players | 19/0 | 13.1 ± 1.3 | ①② | High-intensity interval training | NA | 30s | No |
| ANDREW M. EDWARDS | 2011 | SS ri (n=11)  Fixed ri (n=11) | Trained men | 11/0 | 27±6.9 | ①③ | High-intensity interval training | NA | Rest-to-exercise ratio 1:1 | Yes |
| Eyal Colorni | 2023 | SS ri (n=24)  Fixed ri (n=24) | Amateur cyclist | 24/0 | 36.6±7.2 | ①② | High-intensity interval training | 50 | 90s | No |
| Diogo Correia Cardozo | 2021 | SS ri (n=16)  Fixed1 ri(n=16)  Fixed2 ri (n=16) | Youth Athletes | 16/0 | 19.8 ± 0.9 | ① | Resistance training | 50%–70% 1RM | 1 min  3 min | No |
| Callum G. Brownstein | 2018 | SS ri (n=20)  Fixed ri (n=20) | Youth athletes | 20/0 | NA | ① | High-intensity interval training | NA | 30s | No |
| Asaf Ben-Ari | 2025 | SS ri (n=20)  Fixed ri (n=20) | Professional female soccer player | 0/24 | 22.0 ± 3.8 | ①② | High-intensity interval training | NA | 2 min | No |
| Cristiano Behenck | 2022 | SS ri (n=17)  Fixed1 ri (n=17)  Fixed2 ri (n=17)  Fixed3 ri (n=17) | Army personnel | 17/0 | 21.5±2.1 | ① | Resistance training | 50% 1RM | 1 min  2 min  3 min | No |
| Diego A. Alonso-Aubin | 2024 | SS ri (n=20)  Fixed resistance (n=20) | With resistance training  Adult men | 13/0 | 26.71 ± 3.94 | ①② | Resistance training | 80% 1RM | 2 min | No |

*Note:***SS,** Self-selected interval time; **Fixed,** Fixed interval time;**①** ,Sports Performance Outcome;②, Heart Rate Outcome;③, Blood lactate Outcome
