# Supplementary material for: Systematic review and meta-analysis of the acute effects of self-selected rest intervals on exercise performance maintenance, lactate levels, and heart rate
Source: PLoS One. 2026 Jul 24;21(7):e0354594. doi: 10.1371/journal.pone.0354594 (PMC13399479; doi:10.1371/journal.pone.0354594)
Supplement: S4 Appendix — (DOCX) [file pone.0354594.s004.docx]

**Electronic Supplementary Material Appendix S4 (Subgroup Analysis Table for Heart Rate and Blood Lactate)**

| Subgroup | Hedge’sg | k | p_d_ | p_b_ | lower_CI | upper_CI | CI | I^2^ |
| --- | --- | --- | --- | --- | --- | --- | --- | --- |
| Aid |  |  |  | 0.94 |  |  |  |  |
| No | -0.0035 | 7 | 0.98 |  | -0.35 | 0.34 | (-0.35,0.34) | 42% |
| Yes | -0.06 | 5 | 0.82 |  | -0.59 | 0.47 | (-0.59,0.47) | 58% |
| Training Level |  |  |  | 0.35 |  |  |  |  |
| Althlete | 0.2 | 32 | 0.19 |  | -0.1 | 0.51 | (-0.10,0.51) | 26% |
| Non-althlete | -0.18 | 15 | 0.34 |  | -0.57 | 0.2 | (-0.57,0.20) | 46% |
| Age |  |  |  | 0.46 |  |  |  |  |
| Adolescent | 0.22 | 3 | 0.6 |  | -0.61 | 1.06 | (-0.61,1.06) | 67% |
| Adult | -0.09 | 44 | 0.21 |  | 0.38 | 0.19 | (-0.17,0.76) | 33% |

| Subgroup | Hedge’sg | k | pd | pb | lower_CI | upper_CI | CI | I^2^ |
| --- | --- | --- | --- | --- | --- | --- | --- | --- |
| Training Level |  |  |  | 0.53 |  |  |  |  |
| Althlete | -0.53 | 3 | 0.31 |  | -2.25 | 1.19 | (-2.25,1.19) | 57% |
| Non-althlete | -0.032 | 4 | 0.89 |  | -0.72 | 0.65 | (-0.72,0.65) | 0% |
| Gender |  |  |  | 0.29 |  |  |  |  |
| Male | -0.18 | 5 | 0.41 |  | -0.85 | 0.47 | (-0.85,0.47) | 26% |
| Female | -1.03 | 2 | 0.01 |  | -1.89 | -0.18 | (-1.89,-0.18) | 0% |

***Notes: k*,** the total number of effects included in the pooled effect size; ***Hedge's g***, the effect size indicators used in the pooled; ***95%CI***, 95% confidence interval; ***Pb***, statistically significant P values for pooled effect between moderator; ***Pd***, statistically significant P values or specific pooled effect of moderator; ***I*^2^**, quantitative indicators of heterogeneity.
