# Supplementary material for: Systematic review and meta-analysis of the acute effects of self-selected rest intervals on exercise performance maintenance, lactate levels, and heart rate
Source: PLoS One. 2026 Jul 24;21(7):e0354594. doi: 10.1371/journal.pone.0354594 (PMC13399479; doi:10.1371/journal.pone.0354594)

**Electronic Supplementary Material Appendix S5 (Sensitivity Analysis)**

Sensitivity Analysis of Sports Performance


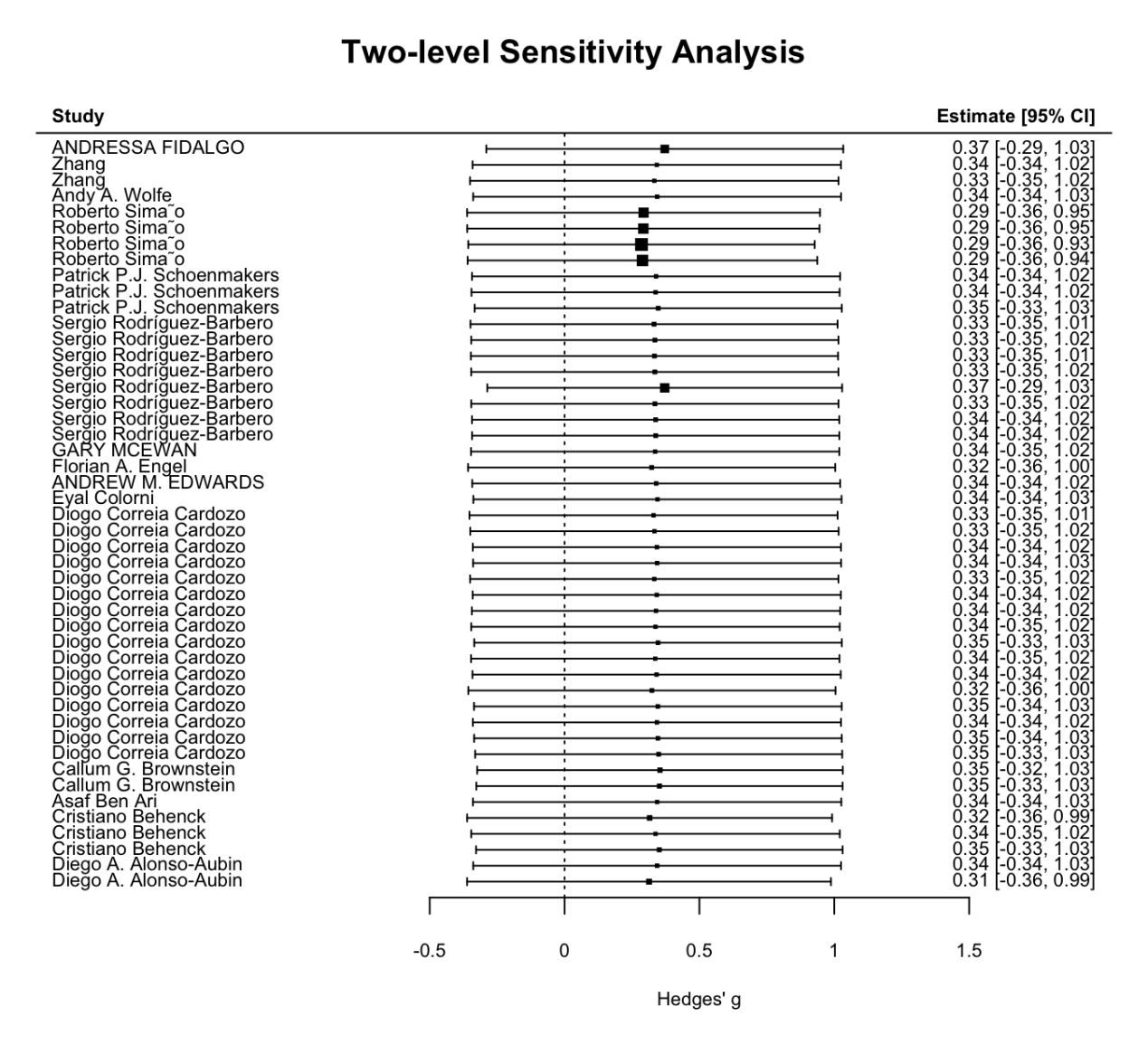


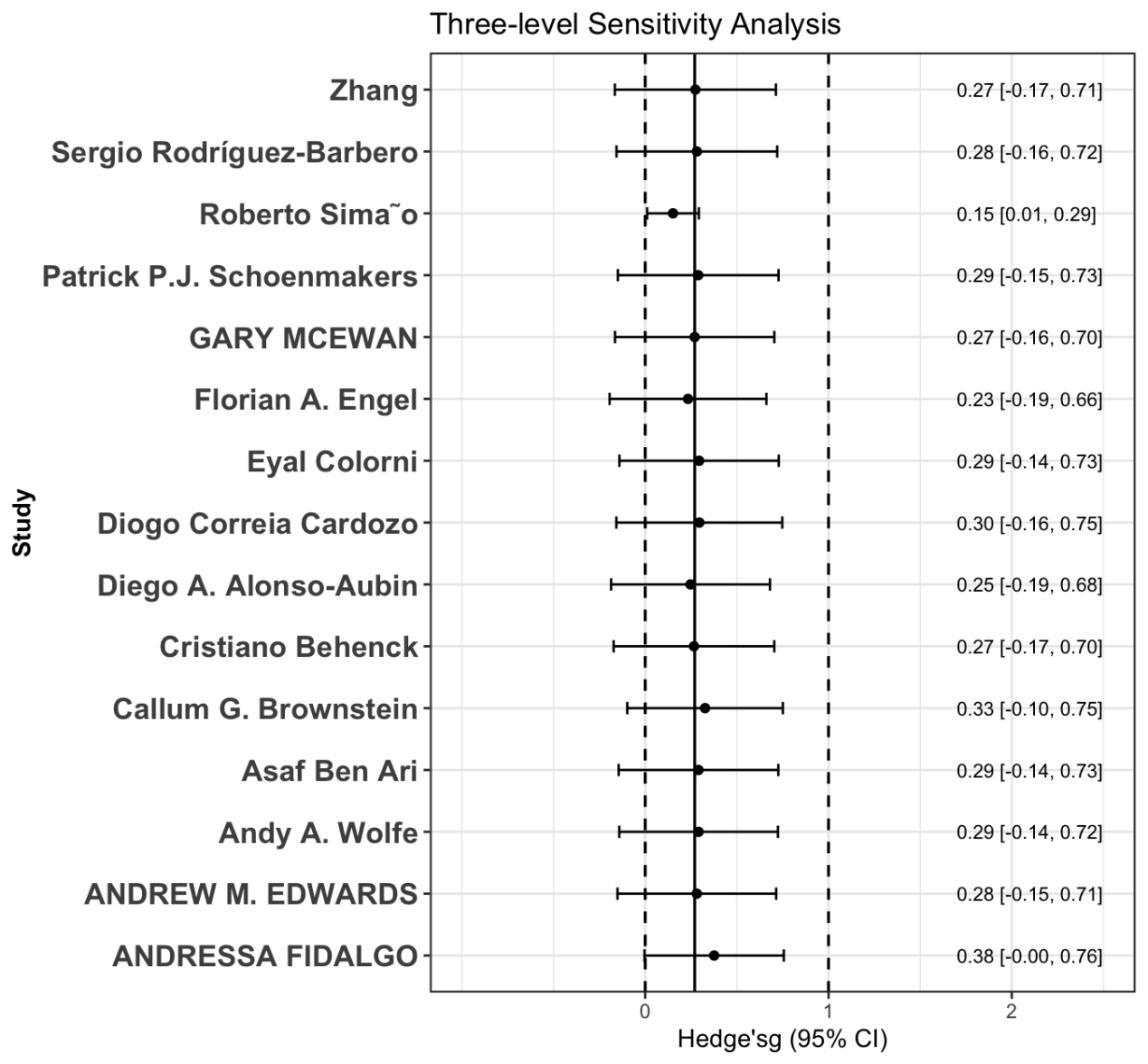


Sensitivity Analysis of Heart Rate
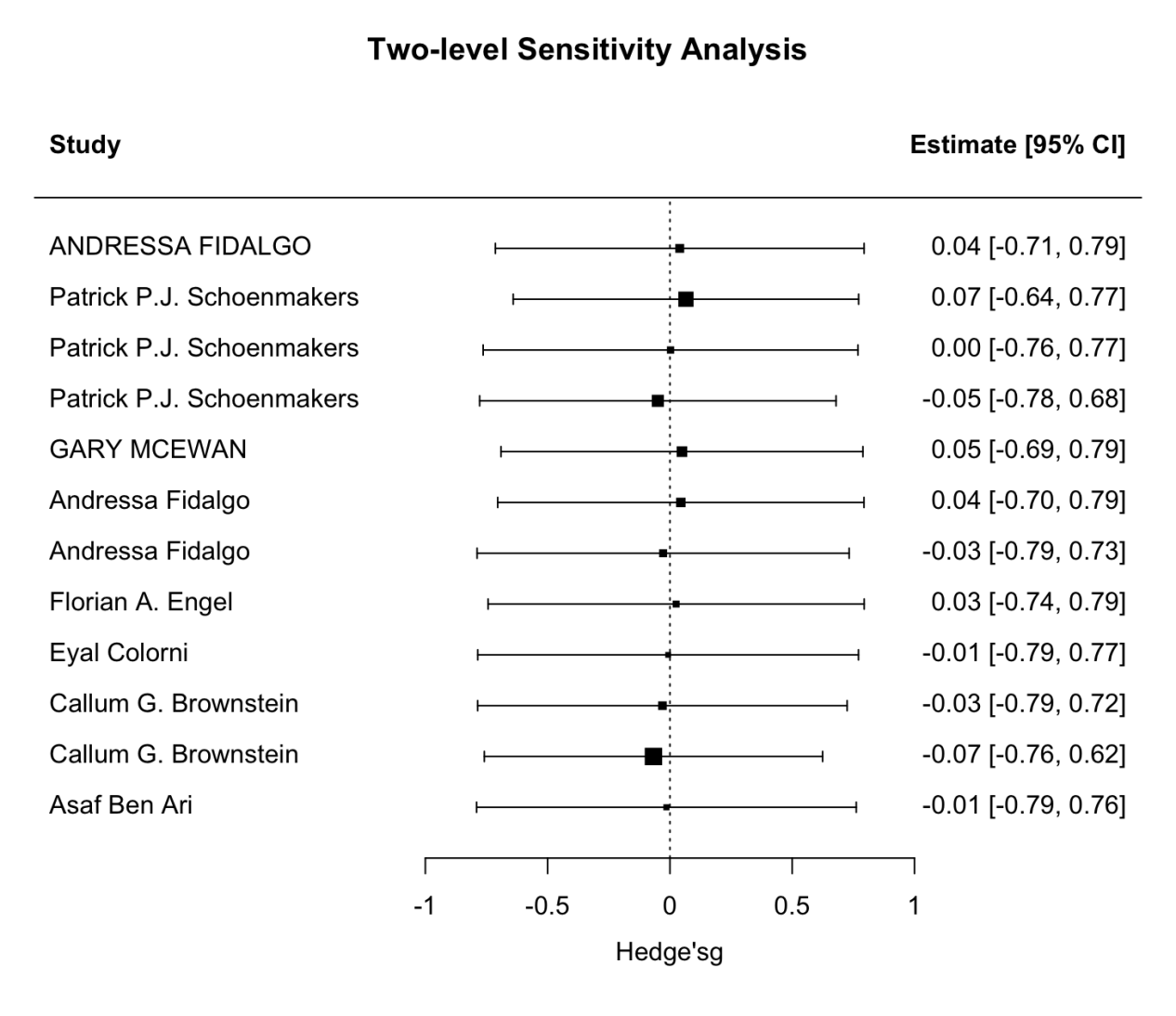


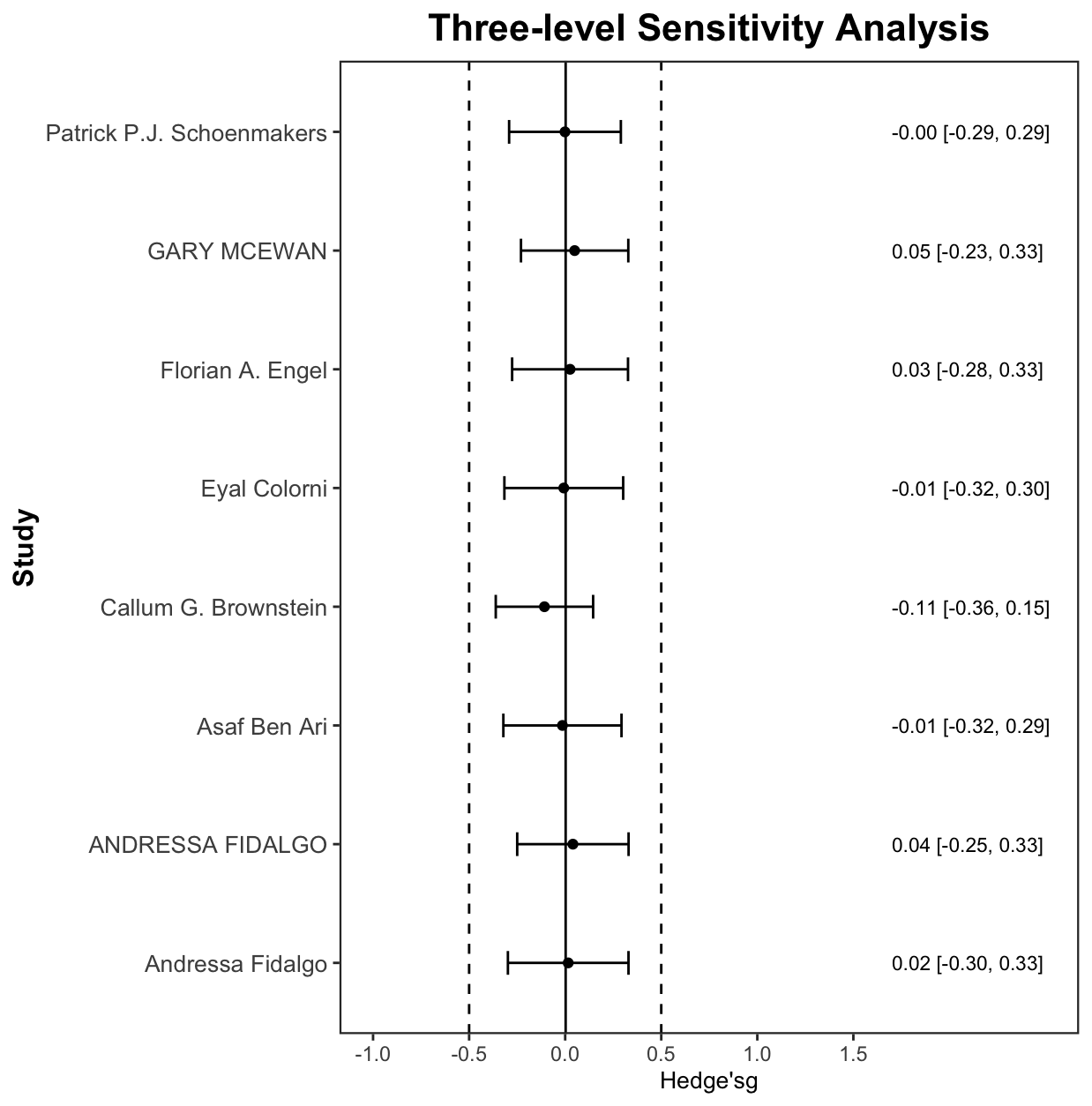


Sensitivity Analysis of the Serial Blood Lactate Method


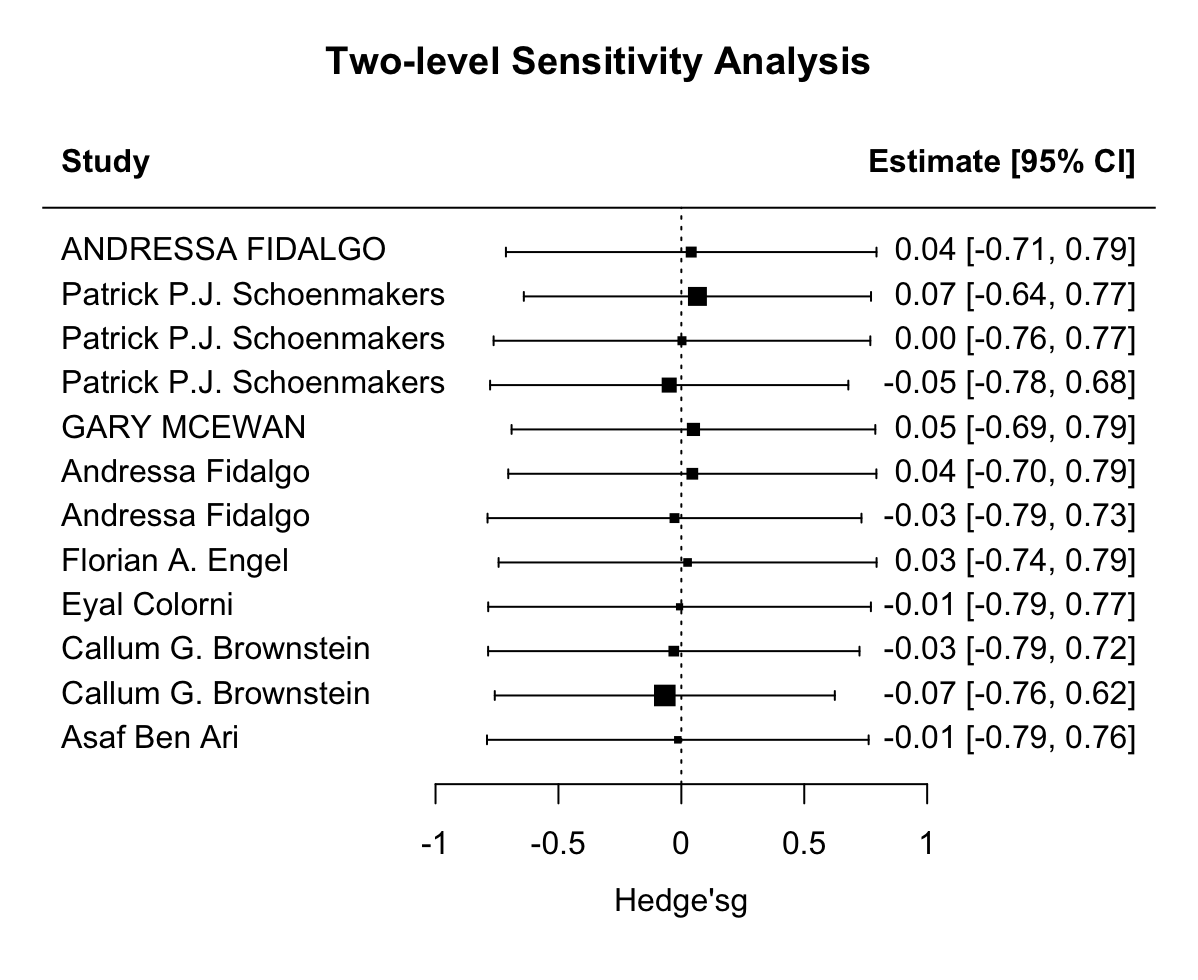


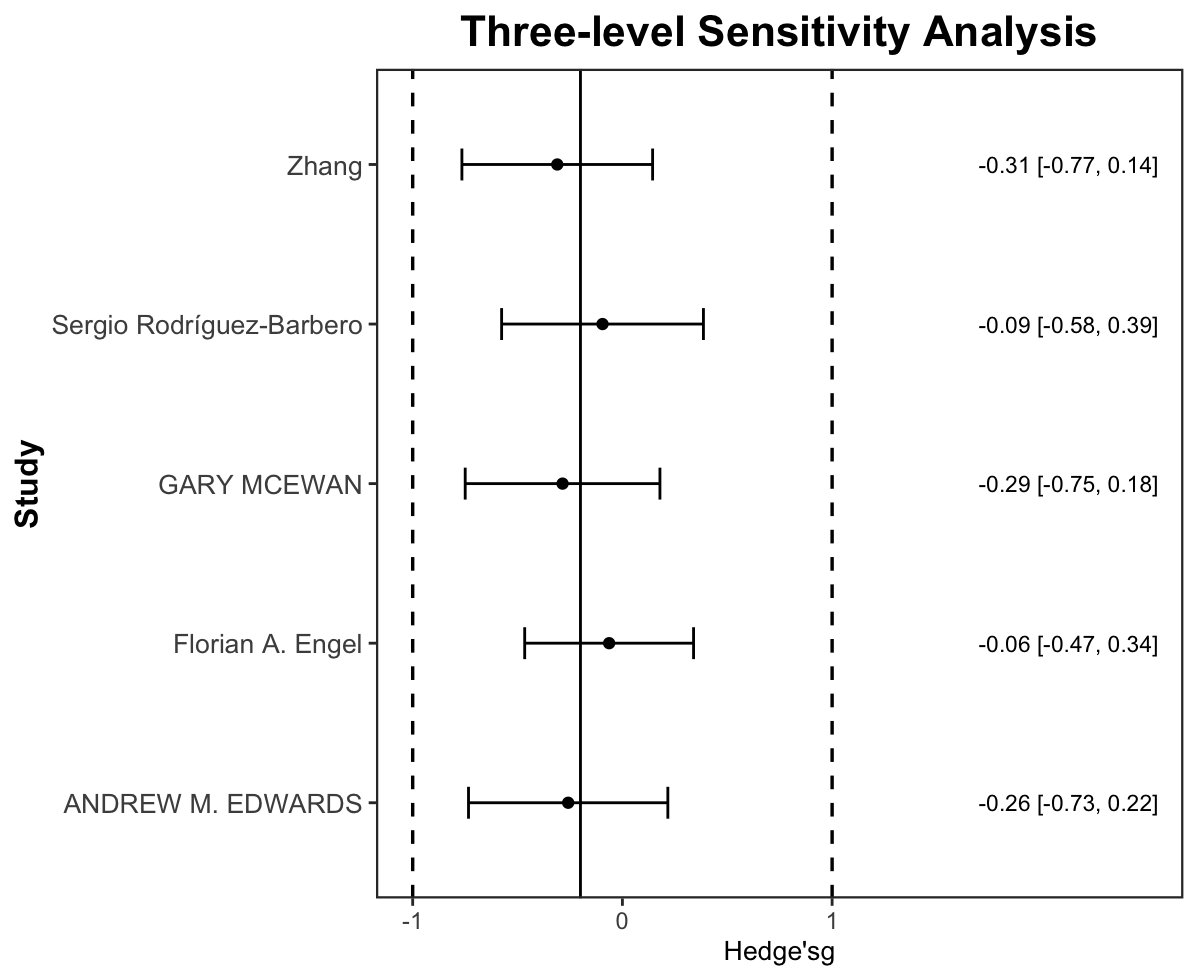

Supplement: S5 Appendix — (DOCX) [file pone.0354594.s005.docx]
