# Supplementary material for: Systematic review and meta-analysis of the acute effects of self-selected rest intervals on exercise performance maintenance, lactate levels, and heart rate
Source: PLoS One. 2026 Jul 24;21(7):e0354594. doi: 10.1371/journal.pone.0354594 (PMC13399479; doi:10.1371/journal.pone.0354594)
Supplement: S6 Appendix — (DOCX) [file pone.0354594.s006.docx]

**Electronic Supplementary Material Appendix S6 (Robust variance estimation)**

| r=0.6 |  |  |  |  |  |  |  |  |  |
| --- | --- | --- | --- | --- | --- | --- | --- | --- | --- |
|  |  |  |  |  |  |  |  |  |  |
| Aggregated study effects | Hedge'sg | **Hedge'sg-Adjust** | P | **P-Adjust** | lower_CI | upper_CI | **lower_CI-Adjust** | **upper_CI-Adjust** |  |
|  | 0.3 | **0.302** | 0.19 | **0.16** | -0.14 | 0.69 | **-0.14** | **0.74** |  |
|  |  |  |  |  |  |  |  |  |  |
| Subgroup | Hedge'sg | **Hedge'sg-Adjust** | k | P | **P-Adjust** | lower_CI | upper_CI | **lower_CI-Adjust** | **upper_CI-Adjust** |
| Aid |  |  |  |  |  |  |  |  |  |
| No | 0.32 | **0.31** | 10 | 0.21 | **0.24** | -0.18 | 0.82 | **-0.24** | **0.88** |
| Yes | 0.08 | **0.07** | 31 | 0.87 | **0.09** | -0.42 | 0.57 | **-0.03** | **0.18** |
| Gender |  |  |  |  |  |  |  |  |  |
| Fixed | 0.2 | **0.2053** | 3 | 0.4 | **0.29** | -0.63 | 1.04 | **-1.11** | **1.52** |
| Male | 0.35 | **0.3358** | 34 | 0.21 | **0.144** | -0.21 | 0.92 | **-0.23** | **0.9** |
| Female | -0.38 | **-0.2701** | 4 | 0.55 | **0.3264** | -2.04 | 1.28 | **-2.2018** | **1.6617** |
| Training Level |  |  |  |  |  |  |  |  |  |
| Althlete | 0.17 | **0.1648** | 31 | 0.02 | **0.0274** | 0.01 | 0.31 | **0.03** | **0.3** |
| Non-althlete | 0.36 | **0.36** | 10 | 0.34 | **0.37** | -0.45 | 1.18 | **-0.54** | **1.27** |
| Age |  |  |  |  |  |  |  |  |  |
| Adolescent | 0.07 | **0.2** | 3 | 0.74 | **0.817** | -1.06 | 1.48 | **-8.04** | **8.46** |
| Adult | 0.29 | **0.28** | 38 | 0.21 | **0.184** | -0.17 | 0.76 | **-0.21** | **0.78** |
| Outcome |  |  |  |  |  |  |  |  |  |
| Oxy patience | 0.04 | **0.15** | 13 | 0.86 | **0.04** | -0.56 | 0.66 | **0** | **0.29** |
| Anaerobic power | 0.63 | **0.04** | 1 | 0.22 | **0.49** | -0.42 | 1.7 | **-0.5** | **0.59** |
| Muscle patience | 0.61 | **0.61** | 21 | 0.24 | **0.29** | -0.41 | 1.63 | **-0.73** | **1.95** |
| Muscle power | 0.1 | **0.09** | 6 | 0.7 | **0.7** | -0.49 | 0.7 | **-0.52** | **0.71** |

| r=0.8 |  |  |  |  |  |  |  |  |  |
| --- | --- | --- | --- | --- | --- | --- | --- | --- | --- |
|  |  |  |  |  |  |  |  |  |  |
| Aggregated study effects | Hedge'sg | **Hedge'sg-Adjust** | P | **P-Adjust** | lower_CI | upper_CI | **lower_CI-Adjust** | **upper_CI-Adjust** |  |
|  | 0.3 | **0.32** | 0.19 | **0.14** | -0.14 | 0.69 | **-0.12** | **0.77** |  |
|  |  |  |  |  |  |  |  |  |  |
| Subgroup | Hedge'sg | **Hedge'sg-Adjust** | k | P | **P-Adjust** | lower_CI | upper_CI | **lower_CI-Adjust** | **upper_CI-Adjust** |
| Aid |  |  |  |  |  |  |  |  |  |
| No | 0.32 | **0.31** | 10 | 0.21 | **0.24** | -0.18 | 0.82 | **-0.24** | **0.88** |
| Yes | 0.08 | **0.07** | 31 | 0.87 | **0.09** | -0.42 | 0.57 | **-0.03** | **0.18** |
| Gender |  |  |  |  |  |  |  |  |  |
| Fixed | 0.2 | **0.2** | 3 | 0.28 | **0.29** | -0.63 | 1.04 | **-1.11** | **1.52** |
| Male | 0.35 | **0.33** | 34 | 0.19 | **0.22** | -0.21 | 0.92 | **-0.23** | **0.9** |
| Female | -0.38 | **-0.27** | 4 | 0.32 | **0.403** | -2.04 | 1.28 | **-2.2** | **1.66** |
| Training Level |  |  |  |  |  |  |  |  |  |
| Althlete | 0.17 | **0.16** | 31 | 0.02 | **0.02** | 0.01 | 0.31 | **0.02** | **0.3** |
| Non-althlete | 0.36 | **0.36** | 10 | 0.34 | **0.37** | -0.45 | 1.18 | **-0.54** | **1.27** |
| Age |  |  |  |  |  |  |  |  |  |
| Adolescent | 0.07 | **0.2** | 3 | 0.74 | **0.8** | -1.06 | 1.48 | **-8.04** | **8.46** |
| Adult | 0.29 | **0.28** | 38 | 0.21 | **0.23** | -0.17 | 0.76 | **-0.21** | **0.78** |
| Outcome |  |  |  |  |  |  |  |  |  |
| Oxy patience | 0.04 | **0.15** | 13 | 0.3 | **0.04** | -0.56 | 0.66 | **0** | **0.29** |
| Anaerobic power | 0.63 | **0.04** | 1 | 0.8 | **0.556** | -0.42 | 1.7 | **-0.5** | **0.59** |
| Muscle patience | 0.61 | **0.61** | 21 | 0.24 | **0.29** | -0.41 | 1.63 | **-0.73** | **1.95** |
| Muscle power | 0.1 | **0.09** | 6 | 0.7 | **0.7** | -0.49 | 0.7 | **-0.52** | **0.71** |

| r=0.4 |  |  |  |  |  |  |  |  |  |
| --- | --- | --- | --- | --- | --- | --- | --- | --- | --- |
|  |  |  |  |  |  |  |  |  |  |
| Aggregated study effects | Hedge'sg | **Hedge'sg-Adjust** | P | **P-Adjust** | lower_CI | upper_CI | **lower_CI-Adjust** | **upper_CI-Adjust** |  |
|  | 0.3 | **0.289** | 0.19 | **0.14** | -0.14 | 0.69 | **-0.15** | **0.729** |  |
|  |  |  |  |  |  |  |  |  |  |
| Subgroup | Hedge'sg | **Hedge'sg-Adjust** | k | P | **P-Adjust** | lower_CI | upper_CI | **lower_CI-Adjust** | **upper_CI-Adjust** |
| Aid |  |  |  |  |  |  |  |  |  |
| No | 0.32 | **0.31** | 10 | 0.21 | **0.24** | -0.18 | 0.82 | **-0.24** | **0.88** |
| Yes | 0.08 | **0.07** | 31 | 0.87 | **0.09** | -0.42 | 0.57 | **-0.03** | **0.18** |
| Gender |  |  |  |  |  |  |  |  |  |
| Fixed | 0.2 | **0.2** | 3 | 0.28 | **0.29** | -0.63 | 1.04 | **-1.11** | **1.52** |
| Male | 0.35 | **0.33** | 34 | 0.19 | **0.22** | -0.21 | 0.92 | **-0.23** | **0.9** |
| Female | -0.38 | **-0.27** | 4 | 0.32 | **0.4** | -2.04 | 1.28 | **-2.2** | **1.21** |
| Training Level |  |  |  |  |  |  |  |  |  |
| Althlete | 0.17 | 0.16 | 31 | 0.02 | **0.02** | 0.01 | 0.31 | **0.02** | **0.3** |
| Non-althlete | 0.36 | 0.37 | 10 | 0.34 | **0.37** | -0.45 | 1.18 | **-0.54** | **1.27** |
| Age |  |  |  |  |  |  |  |  |  |
| Adolescent | 0.07 | 0.2 | 3 | 0.74 | **0.8** | -1.06 | 1.48 | **-8.04** | **8.46** |
| Adult | 0.29 | 0.28 | 38 | 0.21 | **0.23** | -0.17 | 0.76 | **-0.21** | **0.78** |
| Outcome |  |  |  |  |  |  |  |  |  |
| Oxy patience | 0.04 | 0.15 | 13 | 0.3 | **0.04** | -0.56 | 0.66 | **0** | **0.29** |
| Anaerobic power | 0.63 | 0.04 | 1 | 0.8 | **0.49** | -0.42 | 1.7 | **-1.0391** | **0.59** |
| Muscle patience | 0.61 | 0.61 | 21 | 0.24 | **0.29** | -0.41 | 1.63 | **-0.73** | **1.95** |
| Muscle power | 0.1 | 0.09 | 6 | 0.7 | **0.7** | -0.49 | 0.7 | **-0.52** | **0.71** |

|  |  |  |  |  |  |  |  |  |  |
| --- | --- | --- | --- | --- | --- | --- | --- | --- | --- |
|  |  |  |  |  |  |  |  |  |  |
|  |  |  |  |  |  |  |  |  |  |
|  |  |  |  |  |  |  |  |  |  |
|  |  |  |  |  |  |  |  |  |  |
| Estimation of heart rate variability |  |  |  |  |  |  |  |  |  |
| \| r=0.6 \|  \|  \|  \|  \|  \|  \|  \|  \|  \| \| --- \| --- \| --- \| --- \| --- \| --- \| --- \| --- \| --- \| --- \| \|  \|  \|  \|  \|  \|  \|  \|  \|  \|  \| \| Aggregated study effects \| Hedge'sg \| **Hedge'sg-Adjust** \| P \| **P-Adjust** \| lower_CI \| upper_CI \| **lower_CI-Adjust** \| **upper_CI-Adjust** \|  \| \|  \| 0.0024 \| **0.0024** \| 0.98 \| 0.98 \| -0.31 \| 0.31 \| **-0.29** \| **0.3** \|  \| \|  \|  \|  \|  \|  \|  \|  \|  \|  \|  \| \| Subgroup \| Hedges \| **Hedge'sg-Adjust** \| k \| P \| **P-Adjust** \| lower_CI \| upper_CI \| **lower_CI-Adjust** \| **upper_CI-Adjust** \| \| Aid \|  \|  \|  \|  \|  \|  \|  \|  \|  \| \| No \| -0.0035 \| **-0.0035** \| 7 \| 0.98 \| **0.98** \| -0.35 \| 0.34 \| **-0.46** \| **0.45** \| \| Yes \| -0.06 \| **-0.06** \| 5 \| 0.82 \| **0.02** \| -0.59 \| 0.47 \| **-0.08** \| **-0.03** \| \| Training Level \|  \|  \|  \|  \|  \|  \|  \|  \|  \| \| Althlete \| 0.2 \| **0.18** \| 5 \| 0.37 \| **0.29** \| -0.1 \| 0.51 \| **-0.38** \| **0.75** \| \| Non-althlete \| -0.18 \| **0.3358** \| 7 \| 0.34 \| **0.16** \| -0.57 \| 0.2 \| **-0.51** \| **0.14** \| \| Age \|  \| **-0.2701** \|  \|  \| **0.3264** \|  \|  \|  \|  \| \| Adolescent \| 0.22 \| **0.23** \| 3 \| 0.6 \| **0.69** \| -0.61 \| 1.06 \| **-5.23** \| **5.68** \| \| Adult \| -0.09 \| **-0.09** \| 9 \| 0.21 \| **0.28** \| 0.38 \| 0.19 \| **-0.3** \| **0.11** \| |  |  |  |  |  |  |  |  |  |
|  |  |  |  |  |  |  |  |  |  |
|  |  |  |  |  |  |  |  |  |  |
| \| r=0.8 \|  \|  \|  \|  \|  \|  \|  \|  \|  \| \| --- \| --- \| --- \| --- \| --- \| --- \| --- \| --- \| --- \| --- \| \|  \|  \|  \|  \|  \|  \|  \|  \|  \|  \| \| Aggregated study effects \| Hedge'sg \| **Hedge'sg-Adjust** \| P \| **P-Adjust** \| lower_CI \| upper_CI \| **lower_CI-Adjust** \| **upper_CI-Adjust** \|  \| \|  \| 0.0024 \| **0.0024** \| 0.98 \| 0.98 \| -0.31 \| 0.31 \| -0.29 \| 0.3 \|  \| \|  \|  \|  \|  \|  \|  \|  \|  \|  \|  \| \| Subgroup \| Hedges \| **Hedge'sg-Adjust** \| k \| P \| **P-Adjust** \| lower_CI \| upper_CI \| **lower_CI-Adjust** \| **upper_CI-Adjust** \| \| Aid \|  \|  \|  \|  \|  \|  \|  \|  \|  \| \| No \| -0.0035 \| **-0.0035** \| 7 \| 0.98 \| **0.98** \| -0.35 \| 0.34 \| **-0.46** \| **0.45** \| \| Yes \| -0.06 \| **-0.06** \| 5 \| 0.82 \| **0.02** \| -0.59 \| 0.47 \| **-0.08** \| **-0.03** \| \| Training Level \|  \|  \|  \|  \|  \|  \|  \|  \|  \| \| Althlete \| 0.2 \| **0.18** \| 5 \| 0.37 \| **0.37** \| -0.1 \| 0.51 \| **-0.38** \| **0.75** \| \| Non-althlete \| -0.18 \| **0.3358** \| 7 \| 0.34 \| **0.16** \| -0.57 \| 0.2 \| **-0.51** \| **0.14** \| \| Age \|  \| **-0.2701** \|  \|  \| **0.32** \|  \|  \|  \|  \| \| Adolescent \| 0.22 \| **0.23** \| 3 \| 0.6 \| **0.69** \| -0.61 \| 1.06 \| **-5.23** \| **5.68** \| \| Adult \| -0.09 \| **-0.09** \| 9 \| 0.21 \| **0.28** \| 0.38 \| 0.19 \| **-0.3** \| **0.11** \| |  |  |  |  |  |  |  |  |  |
|  |  |  |  |  |  |  |  |  |  |
| \| r=0.4 \|  \|  \|  \|  \|  \|  \|  \|  \|  \| \| --- \| --- \| --- \| --- \| --- \| --- \| --- \| --- \| --- \| --- \| \|  \|  \|  \|  \|  \|  \|  \|  \|  \|  \| \| Aggregated study effects \| Hedge'sg \| **Hedge'sg-Adjust** \| P \| **P-Adjust** \| lower_CI \| upper_CI \| **lower_CI-Adjust** \| **upper_CI-Adjust** \|  \| \|  \| 0.0024 \| **0.0024** \| 0.98 \| 0.98 \| -0.31 \| 0.31 \| -0.29 \| 0.3 \|  \| \|  \|  \|  \|  \|  \|  \|  \|  \|  \|  \| \| Subgroup \| Hedges \| **Hedge'sg-Adjust** \| k \| P \| **P-Adjust** \| lower_CI \| upper_CI \| **lower_CI-Adjust** \| **upper_CI-Adjust** \| \| Aid \|  \|  \|  \|  \|  \|  \|  \|  \|  \| \| No \| -0.0035 \| **-0.0035** \| 7 \| 0.98 \| **0.98** \| -0.35 \| 0.34 \| **-0.46** \| **0.45** \| \| Yes \| -0.06 \| **-0.06** \| 5 \| 0.82 \| **0.02** \| -0.59 \| 0.47 \| **-0.08** \| **-0.03** \| \| Training Level \|  \|  \|  \|  \|  \|  \|  \|  \|  \| \| Althlete \| 0.2 \| **0.18** \| 5 \| 0.37 \| **0.37** \| -0.1 \| 0.51 \| **-0.38** \| **0.75** \| \| Non-althlete \| -0.18 \| **0.3358** \| 7 \| 0.34 \| **0.16** \| -0.57 \| 0.2 \| **-0.51** \| **0.14** \| \| Age \|  \| **-0.2701** \|  \|  \| **0.3264** \|  \|  \|  \|  \| \| Adolescent \| 0.22 \| **0.23** \| 3 \| 0.6 \| **0.69** \| -0.61 \| 1.06 \| **-5.23** \| **5.68** \| \| Adult \| -0.09 \| **-0.09** \| 9 \| 0.21 \| **0.28** \| 0.38 \| 0.19 \| **-0.3** \| **0.11** \| |  |  |  |  |  |  |  |  |  |
| Estimation of the variance of blood lactate stability   \| r=0.6 \|  \|  \|  \|  \|  \|  \|  \|  \|  \| \| --- \| --- \| --- \| --- \| --- \| --- \| --- \| --- \| --- \| --- \| \|  \|  \|  \|  \|  \|  \|  \|  \|  \|  \| \| Aggregated study effects \| Hedge'sg \| **Hedge'sg-Adjust** \| P \| **P-Adjust** \| lower_CI \| upper_CI \| **lower_CI-Adjust** \| **upper_CI-Adjust** \|  \| \|  \| -0.2 \| **-0.2** \| 0.36 \| 0.38 \| -0.69 \| 0.29 \| -0.77 \| 0.37 \|  \| \|  \|  \|  \|  \|  \|  \|  \|  \|  \|  \| \| Subgroup \| Hedges \| **Hedge'sg-Adjust** \| k \| P \| **P-Adjust** \| lower_CI \| upper_CI \| **lower_CI-Adjust** \| **upper_CI-Adjust** \| \| Gender \|  \|  \|  \|  \|  \|  \|  \|  \|  \| \| Male \| -0.18 \| **-0.18** \| 5 \| 0.41 \| **0.48** \| -0.63 \| 0.25 \| **-0.94** \| **0.57** \| \| Female \| -1.03 \| **NA** \| 2 \| 0.01 \| **NA** \| -1.89 \| -0.18 \| **NA** \| **NA** \| \| Training Level \|  \|  \|  \|  \|  \|  \|  \|  \|  \| \| Althlete \| -0.42 \| **-0.42** \| 4 \| 0.18 \| **0.26** \| -1.03 \| 0.19 \| **-1.61** \| **0.76** \| \| Non-althlete \| -0.03 \| **-0.03** \| 4 \| 0.89 \| **0.16** \| -0.45 \| 0.39 \| **-0.91** \| **0.85** \| \|  \|  \|  \|  \|  \|  \|  \|  \|  \|  \| \| r=0.8 \|  \|  \|  \|  \|  \|  \|  \|  \|  \| \|  \|  \|  \|  \|  \|  \|  \|  \|  \|  \| \| Aggregated study effects \| Hedge'sg \| **Hedge'sg-Adjust** \| P \| **P-Adjust** \| lower_CI \| upper_CI \| **lower_CI-Adjust** \| **upper_CI-Adjust** \|  \| \|  \| -0.2 \| **-0.2** \| 0.36 \| **0.38** \| -0.69 \| 0.29 \| -0.77 \| 0.37 \|  \| \|  \|  \|  \|  \|  \|  \|  \|  \|  \|  \| \| Subgroup \| Hedges \| **Hedge'sg-Adjust** \| k \| **P** \| **P-Adjust** \| lower_CI \| upper_CI \| **lower_CI-Adjust** \| **upper_CI-Adjust** \| \| Gender \|  \|  \|  \|  \|  \|  \|  \|  \|  \| \| Male \| -0.18 \| **-0.18** \| 5 \| 0.41 \| **0.48** \| -0.63 \| 0.25 \| **-0.94** \| **0.57** \| \| Female \| -1.03 \| **NA** \| 2 \| 0.01 \| **NA** \| -1.89 \| -0.18 \| **NA** \| **NA** \| \| Training Level \|  \|  \|  \|  \|  \|  \|  \|  \|  \| \| Althlete \| -0.42 \| **-0.42** \| 4 \| 0.18 \| **0.26** \| -1.03 \| 0.19 \| **-1.61** \| **0.76** \| \| Non-althlete \| -0.03 \| **-0.03** \| 4 \| 0.89 \| **0.16** \| -0.45 \| 0.39 \| **-0.91** \| **0.85** \| \|  \|  \|  \|  \|  \|  \|  \|  \|  \|  \| \| r=0.4 \|  \|  \|  \|  \|  \|  \|  \|  \|  \| \|  \|  \|  \|  \|  \|  \|  \|  \|  \|  \| \| Aggregated study effects \| Hedge'sg \| **Hedge'sg-Adjust** \| P \| **P-Adjust** \| lower_CI \| upper_CI \| **lower_CI-Adjust** \| **upper_CI-Adjust** \|  \| \|  \| -0.2 \| **-0.2** \| 0.36 \| 0.38 \| -0.69 \| 0.29 \| -0.77 \| 0.37 \|  \| \|  \|  \|  \|  \|  \|  \|  \|  \|  \|  \| \| Subgroup \| Hedges \| **Hedge'sg-Adjust** \| k \| P \| **P-Adjust** \| lower_CI \| upper_CI \| **lower_CI-Adjust** \| **upper_CI-Adjust** \| \| Gender \|  \|  \|  \|  \|  \|  \|  \|  \|  \| \| Male \| -0.18 \| **-0.18** \| 5 \| 0.41 \| **0.48** \| -0.63 \| 0.25 \| **-0.94** \| **0.57** \| \| Female \| -1.03 \| **NA** \| 2 \| 0.01 \| **NA** \| -1.89 \| -0.18 \| **NA** \| **NA** \| \| Training Level \|  \|  \|  \|  \|  \|  \|  \|  \|  \| \| Althlete \| -0.42 \| **-0.42** \| 4 \| 0.18 \| **0.26** \| -1.03 \| 0.19 \| **-1.61** \| **0.76** \| \| Non-althlete \| -0.03 \| **-0.03** \| 4 \| 0.89 \| **0.16** \| -0.45 \| 0.39 \| **-0.91** \| **0.85** \| |  |  |  |  |  |  |  |  |  |
| ***Notes: k*,** the total number of effects included in the pooled effect size; ***Hedge's g***, the effect size indicators used in the pooled; ***95%CI***, 95% confidence interval; ***P***, statistically significant P values or specific pooled effect of moderator;***lower_CI***,lower limit of the confidence interval;***upper_CI***,Upper limit of the confidence interval;***Hedge'sg-Adjust*,**  Effect size measures used in the adjusted，***P-Adjust，***Statistically significant P-values after adjustment, or the specific combined effects of the moderating variables；  ***lower_CI-Adjust*，**Lower limit of the adjusted confidence interval;***upper_CI-Adjust***,Upper limit of the corrected confidence interval. |  |  |  |  |  |  |  |  |  |
