# Supplementary figures and images for: Systematic review and meta-analysis of the acute effects of self-selected rest intervals on exercise performance maintenance, lactate levels, and heart rate
Source: PLoS One. 2026 Jul 24;21(7):e0354594. doi: 10.1371/journal.pone.0354594 (PMC13399479; doi:10.1371/journal.pone.0354594)

| 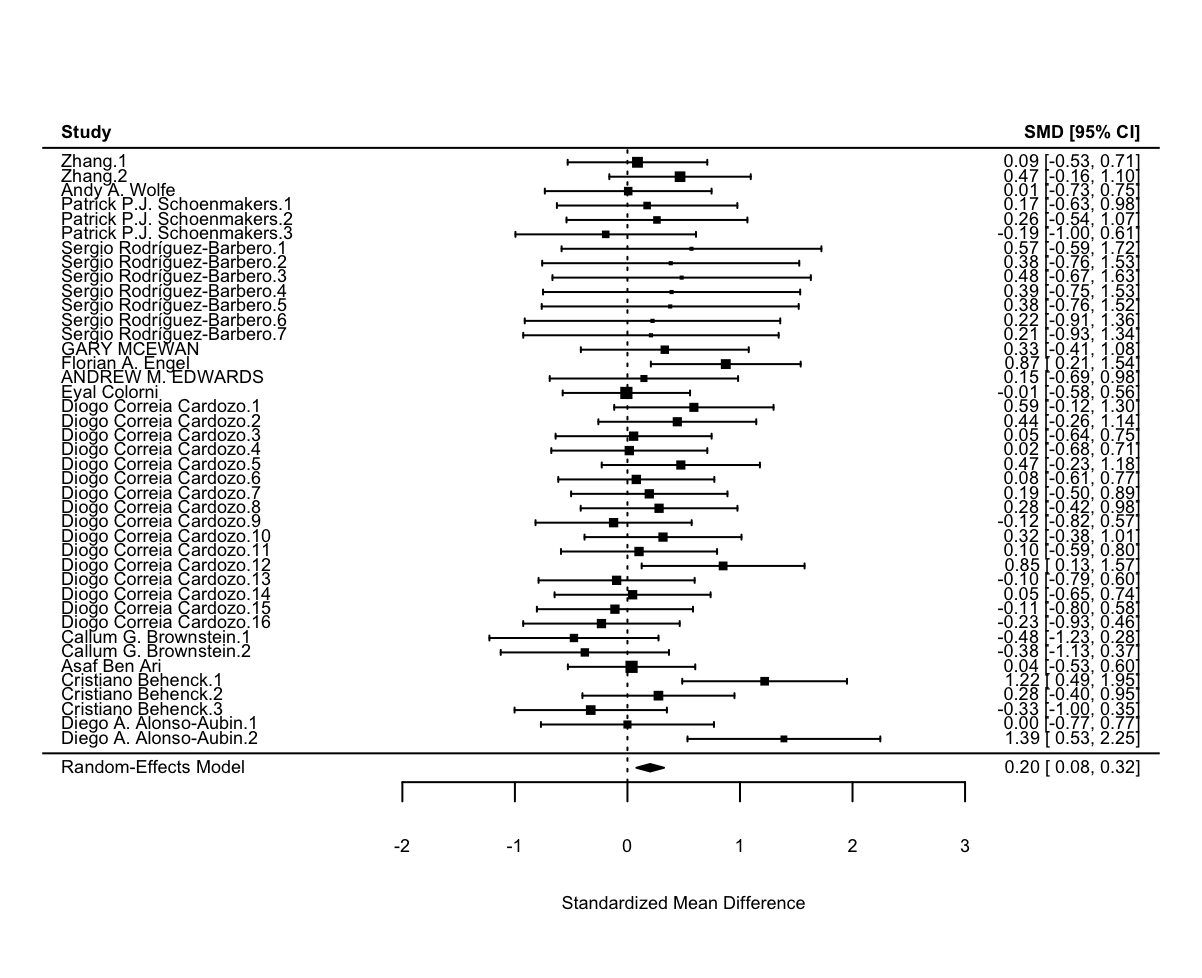**Electronic Supplementary Material Appendix S7 (Main Effects Plot with Outliers Removed)** |
| --- |
|  |
|  |
| 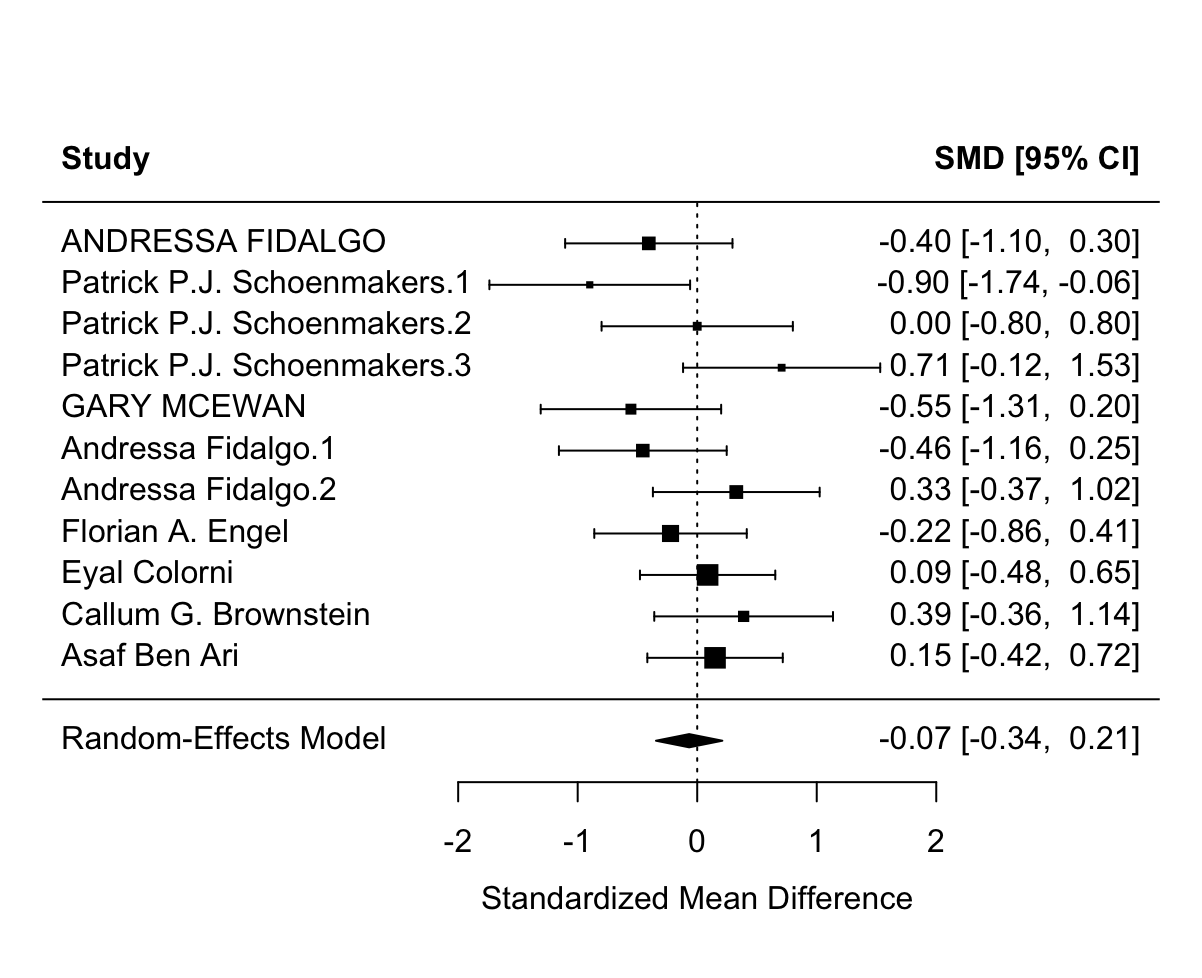 |

Supplement: S7 Appendix — (DOCX) [file pone.0354594.s007.docx]
