# Supplementary material for: Systematic review and meta-analysis of the acute effects of self-selected rest intervals on exercise performance maintenance, lactate levels, and heart rate
Source: PLoS One. 2026 Jul 24;21(7):e0354594. doi: 10.1371/journal.pone.0354594 (PMC13399479; doi:10.1371/journal.pone.0354594)
Supplement: S8 Appendix — (DOCX) [file pone.0354594.s008.docx]

| **Electronic Supplementary Material Appendix S8 (Subgroup Analysis After Outlier Removal)**   \| Subgroup \| Hedges \| CI \| k \| Pd \| P \| I^2^ \| lower_CI \| upper_CI \| \| --- \| --- \| --- \| --- \| --- \| --- \| --- \| --- \| --- \| \| Aid \|  \|  \|  \| <0.01 \|  \|  \|  \|  \| \| No \| 0.22 \| (0.09,0.35) \| 10 \|  \| <0.01 \| 11% \| 0.09 \| 0.35 \| \| Yes \| 0.08 \| (-0.30,0.43) \| 31 \|  \| 0.69 \| 0% \| -0.3 \| 0.43 \| \| Gender \|  \|  \|  \| 0.02 \|  \|  \|  \|  \| \| Fixed \| 0.2 \| (-0.17,0.58) \| 3 \|  \| 0.33 \| 0% \| -0.17 \| 0.58 \| \| Male \| 0.21 \| (0.07,0.35) \| 34 \|  \| <0.01 \| 15% \| 0.07 \| 0.35 \| \| Female \| 0.14 \| (-0.29,0.56) \| 4 \|  \| 0.54 \| 0% \| -0.29 \| 0.56 \| \| Training Level \|  \|  \|  \| <0.01 \|  \|  \|  \|  \| \| Althlete \| 0.19 \| (0.04,0.33) \| 31 \|  \| 0.01 \| 8% \| 0.04 \| 0.33 \| \| Non-althlete \| 0.25 \| (0.01,0.48) \| 10 \|  \| 0.03 \| 0% \| 0.01 \| 0.48 \| \| Age \|  \|  \|  \| <0.01 \|  \|  \|  \|  \| \| Adolescent \| 0.2 \| (-1.06,1.48) \| 3 \|  \| 0.75 \| 86% \| -1.06 \| 1.48 \| \| Adult \| 0.21 \| ( 0.09,0.33) \| 38 \|  \| <0.01 \| 0% \| 0.09 \| 0.33 \| \| Outcome \|  \|  \|  \| 0.09 \|  \|  \|  \|  \| \| Oxy patience \| 0.25 \| (-0.04,0.54) \| 13 \|  \| 0.09 \| 0% \| -0.04 \| 0.54 \| \| Anaerobic power \| 0.04 \| (-0.31,0.4) \| 1 \|  \| 0.87 \| 0% \| -0.31 \| 0.4 \| \| Muscle patience \| 0.4 \| (0.05,0.81) \| 21 \|  \| 0.02 \| 44.00% \| 0.05 \| 0.81 \| \| Muscle power \| 0.09 \| (-0.34,0.50) \| 6 \|  \| 0.68 \| 50% \| -0.34 \| 0.53 \| |
| --- | --- | --- | --- | --- | --- | --- | --- | --- | --- | --- | --- | --- | --- | --- | --- | --- | --- | --- | --- | --- | --- | --- | --- | --- | --- | --- | --- | --- | --- | --- | --- | --- | --- | --- | --- | --- | --- | --- | --- | --- | --- | --- | --- | --- | --- | --- | --- | --- | --- | --- | --- | --- | --- | --- | --- | --- | --- | --- | --- | --- | --- | --- | --- | --- | --- | --- | --- | --- | --- | --- | --- | --- | --- | --- | --- | --- | --- | --- | --- | --- | --- | --- | --- | --- | --- | --- | --- | --- | --- | --- | --- | --- | --- | --- | --- | --- | --- | --- | --- | --- | --- | --- | --- | --- | --- | --- | --- | --- | --- | --- | --- | --- | --- | --- | --- | --- | --- | --- | --- | --- | --- | --- | --- | --- | --- | --- | --- | --- | --- | --- | --- | --- | --- | --- | --- | --- | --- | --- | --- | --- | --- | --- | --- | --- | --- | --- | --- | --- | --- | --- | --- | --- | --- | --- | --- | --- | --- | --- | --- | --- | --- | --- | --- | --- | --- | --- | --- | --- | --- | --- | --- |
