# Supplementary material for: Systematic review and meta-analysis of the acute effects of self-selected rest intervals on exercise performance maintenance, lactate levels, and heart rate
Source: PLoS One. 2026 Jul 24;21(7):e0354594. doi: 10.1371/journal.pone.0354594 (PMC13399479; doi:10.1371/journal.pone.0354594)
Supplement: S9 Appendix — (DOCX) [file pone.0354594.s009.docx]

**Electronic Supplementary Material Appendix S9 (Summary of Evidence Quality)**

| **Outcome** | **K** | **Certainty of Evidence Assessment** | | | | | **Hedge’s g [95% CI] *** | **GRADE†** |
| --- | --- | --- | --- | --- | --- | --- | --- | --- |
|  |  | **Risk of Bias** | **Inconsistency** | **Indirectness** | **Imprecision** | **Others** |  |  |
| Primary Outcome | | | | | | | | |
| Exercise performance | K = 15 | Serious | serious | Not serious | serious | None | 0.28 [-0.13, 0.70] | ⨁◯◯◯ Very low |
| Heart Rate | K = 8 | Serious | Not serious | Not serious | serious | None | 0.0024 [-0.30, 0.31] | ⨁⨁◯◯  Low |
| Blood lactic acid | K = 5 | Serious | Not serious | Not serious | serious | None | -0.20 [-0.69, 0.29] | ⨁⨁◯◯ Low |
| ***Notes: K*:** the total number of effects included in the pooled effect size; ***Publication bias***, represented by Egger test p < 0.05. | | | | | | | | |
| ***:** The effect size (*Hedges’g*) was significant (p < 0.05).  **†** **GRADE Criteria for Certainty of Evidence**：  **High**: Very confident in the estimated effect.  **Moderate**: Moderately confident in the estimated effect.  **Low**: Limited confidence in the estimated effect.  **Very low**: Very limited confidence in the estimated effect | | | | | | | | |
