# Supplementary material for: Systematic review and meta-analysis of the acute effects of self-selected rest intervals on exercise performance maintenance, lactate levels, and heart rate
Source: PLoS One. 2026 Jul 24;21(7):e0354594. doi: 10.1371/journal.pone.0354594 (PMC13399479; doi:10.1371/journal.pone.0354594)
Supplement: S10 Appendix — (DOCX) [file pone.0354594.s010.docx]

**Electronic Supplementary Material Appendix S10 (Regression Analysis with Outlier Removal)**

Regression Analysis with Outlier Removal for Maintaining Athletic Performance


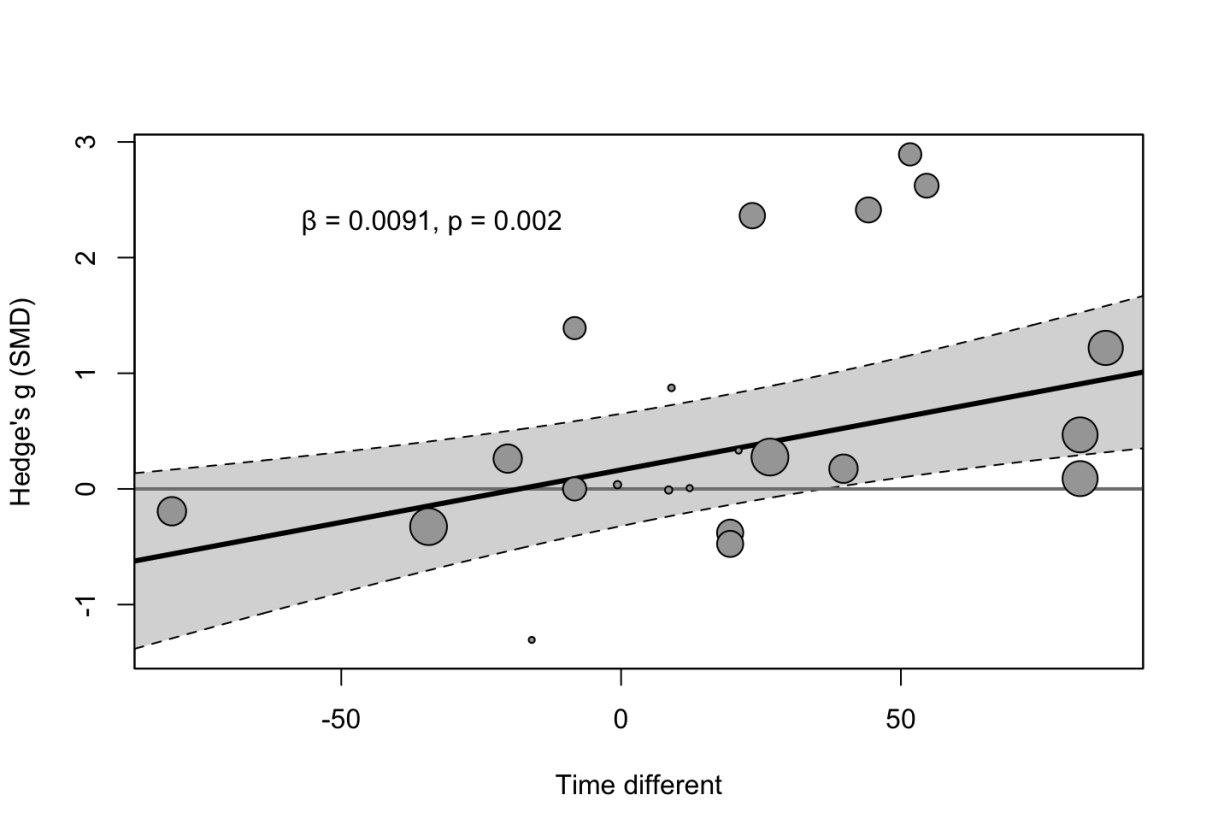

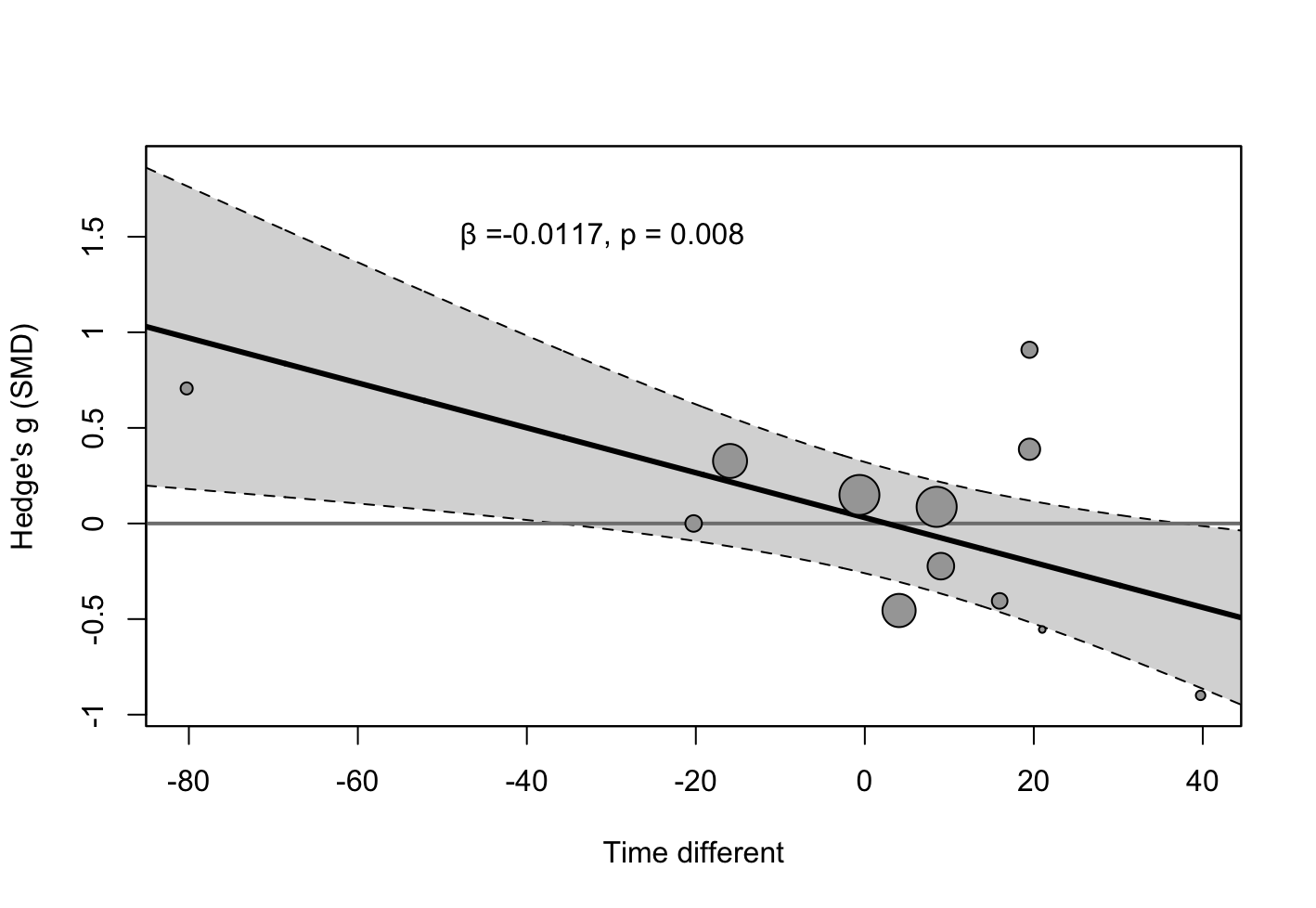


Regression Analysis with Outlier Removal for Heart Rate Data
